# Gammaherpesvirus Alters Alveolar Macrophages According to the Host Genetic Background and Promotes Beneficial Inflammatory Control over Pneumovirus Infection

**DOI:** 10.3390/v14010098

**Published:** 2022-01-06

**Authors:** Gautier Gilliaux, Daniel Desmecht

**Affiliations:** Department of Animal Pathology, FARAH and Faculty of Veterinary Medicine, University of Liège, 4000 Liège, Belgium; ggilliaux@uliege.be

**Keywords:** immunopathogenesis, pneumoviridae, gammaherpesvirus, inflammation, animal models, alveolar macrophages, innate and adaptive immunity

## Abstract

Human respiratory syncytial virus (hRSV) infection brings a wide spectrum of clinical outcomes, from a mild cold to severe bronchiolitis or even acute interstitial pneumonia. Among the known factors influencing this clinical diversity, genetic background has often been mentioned. In parallel, recent evidence has also pointed out that an early infectious experience affects heterologous infections severity. Here, we analyzed the importance of these two host-related factors in shaping the immune response in pneumoviral disease. We show that a prior gammaherpesvirus infection improves, in a genetic background-dependent manner, the immune system response against a subsequent lethal dose of pneumovirus primary infection notably by inducing a systematic expansion of the CD8^+^ bystander cell pool and by modifying the resident alveolar macrophages (AMs) phenotype to induce immediate cyto/chemokinic responses upon pneumovirus exposure, thereby drastically attenuating the host inflammatory response without affecting viral replication. Moreover, we show that these AMs present similar rapid and increased production of neutrophil chemokines both in front of pneumoviral or bacterial challenge, confirming recent studies attributing a critical antibacterial role of primed AMs. These results corroborate other recent studies suggesting that the innate immunity cells are themselves capable of memory, a capacity hitherto reserved for acquired immunity.

## 1. Introduction

Human respiratory syncytial virus (hRSV) infection leads to a wide spectrum of clinical outcomes, from a mild cold to severe bronchiolitis, pneumonia, or even asthma. Nearly all children have been infected with hRSV at least once by two years of age. Approximately 1–2% of them develop a disease that requires hospitalization, which makes it important to identify the key factors underlying the severe clinical forms [1,2,3]. In this area, available studies have focused on socio-demographic and child-related risk factors. The most comprehensive report of the strength of association between various socio-demographic indicators and hRSV-associated severe lower respiratory infection identified eight risk factors: prematurity, low birth weight, being male, siblings, maternal smoking, history of atopy, no breastfeeding, and crowding ≥7 persons in the household [4]. The search for the child-, or host-related factors underlying this clinical diversity has long favored the genetic hypothesis. Studies of otherwise healthy infants and children have identified single nucleotide polymorphisms (SNPs) that are overrepresented in patients hospitalized with hRSV compared to either healthy controls or patients with milder hRSV disease. Most of these severe bronchiolitis-associated SNPs bias the adaptive immunity Th1/Th2 balance toward a Th2-dominated response [5,6,7,8,9,10,11]. At present, however, no decisive association has been demonstrated in a reproducible manner, and hRSV susceptibility/resistance is attributed to a large number of alleles acting in concert at multiple levels within the host [12]. Another factor that could explain the predisposition of some children to the hRSV-induced disease could be related to extrinsic factors affecting the development of the immune system in early life. Indeed, several studies have highlighted that microbiome composition can impact the development of hRSV pathogenesis [13]. In particular, persistent viruses such as gammaherpesviruses have coevolved with their host and appear to broadly influence the composition and function of the immune system [14,15]. Interestingly, while human gammaherpesvirus Epstein-Barr virus (EBV) infection usually occurs during childhood, recent epidemiological surveys highlighted a progressively increased age of seroconversion to EBV in developed countries [16]. Therefore, the sequence following which children encounter gammaherpesviruses and hRSV at an early age might influence the outcome of RSV infections. Though immune memory is a canonical defining feature of the acquired immune system, a series of evidence aggregated over the past decade has pointed out that activation of the innate immune system can result in enhanced responsiveness to subsequent heterologous triggers. Specifically, by demonstrating that an early infectious experience affects how a given host responds to a later heterologous infection, this evidence forged the new biological concept termed “innate immune memory”. This process has been shown to offer either broad benefits for host defense or, on the contrary, detrimental overactivation of the host response [17]. Among this new field of research, “priming” is defined as a stimulus that changes the functional state of cells and their immune status and does not return to basal levels before the second infection. Therefore, the impact of the second challenge in primed cells is synergistic with the original stimuli [18].

In the following study, we compared the respective importance of these two host-related factors, genetic make-up, and immune priming, in shaping the diversity of the host response in hRSV disease. We have used the natural rodent pathogen pneumonia virus of mice (PVM), which is closely related to hRSV [19,20]. While hRSV does not replicate effectively in inbred mouse strains [21], PVM undergoes robust replication and generates severe inflammatory disease in association with cytokine production and prominent leukocyte recruitment to the lungs. As acute PVM infection in mice phenocopies the clinical, histological, and inflammatory responses characteristic of the most severe forms of hRSV infection in human subjects, it is widely regarded as the most relevant model of severe hRSV disease [21,22]. In parallel, we pre-exposed mice to murid herpesvirus 4 (MuHV-4), a gammaherpesvirus already known to exert a long-term effect on lung immune innate cells in an asthma model [15]. Finally, because of the proven association between clinical severity of hRSV disease and alleles controlling the Th1/Th2 balance, we enrolled an outbred mouse strain (CD-1) to represent the intrinsic genetic diversity of the *Mus musculus* species and two inbred strains (C57BL/6 and BALB/c) which are, respectively, prototypical Th1- and Th2-type mouse strains [23,24,25].

In the context of successive inoculations of MuHV-4 and PVM, the study revealed that a past infectious experience on the scenario of subsequent infection is effective in a genetic background-dependent manner. Moreover, the remarkable innate immune priming provided by MuHV-4 consisted of redirecting and modifying the alveolar macrophages (AMs) cyto/chemokine immediate responses upon exposure to PVM, thereby drastically attenuating the host inflammatory response and disease severity.

## 2. Materials and Methods

### 2.1. Design

To examine the effect granted by a first infection with a herpesvirus (priming) on the course of a subsequent, theoretically lethal pneumovirus infection, 36 mice from each of two inbred (BALB/c and C57BL/6) and one outbred (CD-1) strains were inoculated with the pathogenic strain of the mouse pneumovirus (PVM, J3666). Inbred strains BALB/c and C57BL/6 were chosen deliberately because they are reputed to be prototypical of Th2 and Th1 responses, respectively. The outbred strain was enrolled to discriminate a species-typical response from those genetically biased by prolonged inbreeding selection. Within each strain, 3 subsets were examined, which differed according to the priming imposed 28 days before PVM inoculation, either PBS (*n* = 12), live murid herpesvirus-4 (MuHV-4, *n* = 12), or heat-inactivated MuHV-4 (_hi_MuHV-4, *n* = 12). Twenty-eight days after MuHV-4 infection, the first half of each subset’s cohort (*n* = 6) was euthanized, and the second half was inoculated with PVM, then euthanized on day 6 post infection (p.i.) (day 34 post MuHV-4) for autopsy, histological examination, lung cell subsets, viral load, and cytokines/chemokines quantitation. In a side experiment, 6 mice of each cohort were enrolled in a longitudinal study during which clinical scoring, subcutaneous temperature, and body weight values were measured daily, starting 1 day before PVM inoculation and going on up to the point of accepting death as a predictable outcome post inoculation (survival study).

### 2.2. Mice, Virus Infection, and Disease Monitoring

The experiments were conducted with specific pathogen-free 8-wk-old BALB/c, C57BL/6, and CD-1 female mice obtained from Janvier Laboratories (Le Genest Saint Isle, France). Housing, inoculation, data collection, and euthanasia procedures complied with National Institutes of Health guidelines, and the experimental protocol was approved by the Institutional Animal Care and Use Committee of the University of Liège (permits #1651 and #2329).

The MHV-68 strain of MuHV-4 was propagated on BHK-21 cells. Cells and supernatant were harvested at about 4 days post infection (~85% of lysis), and debris was removed by low-speed centrifugation (350× *g*, 15 min, 4 °C). Virions present in the supernatant were harvested by ultracentrifugation (100,000× *g*, 2 h at 4 °C) and banded by isopycnic gradient ultracentrifugation through a 20% to 50% weight/volume potassium tartrate gradient in PBS (100,000× *g*, 2 h at 4 °C). The band containing virions was collected, washed, and concentrated further in PBS by ultracentrifugation (100,000× *g*, 2 h at 4 °C). Finally, the pellet was resuspended in PBS, and virus-enriched preparations were aliquoted and stored at -80 °C. A fraction of these aliquots was heated at 65 °C for 30 min and expected inactivation of MuHV-4 was confirmed by culture on BHK-21 cells. A virulent stock of PVM strain J3666 was obtained by blade homogenization of mouse lungs after serial passages in 8-wk-old mice. The stock was produced in DMEM complemented with 10% FBS, then clarified by centrifugation, filtered, dialyzed, divided into aliquots, and stored in liquid nitrogen prior to use. Infectious titers of both stocks were determined by standard median tissue culture infectious dose (TCID_50_) assay on BHK-21 cells, and prior titration of a series of aliquots drawn at random yielded highly reproducible titers.

The inoculation procedures consisted of instilling 50 μL of vehicle (PBS), heated (_hi_MuHV-4) and live MuHV-4 (1.4 × 10^4^ TCID_50_ in PBS) or PVM (10 or 4 TCID_50_ in PBS) into the nostrils of the anesthetized mouse (ketamine/xylazine, ip) maintained in a vertical position. The PVM dose was intended to examine the lungs at the peak of induced disease, which usually occurs 6 days after inoculation [26]. A previous dose-response study had shown that inocula of 25, 10, and 4 TCID_50_ caused disease in all mice and that no more than 4 TCID_50_ had to be inoculated for maintaining 100% survival 6 days after inoculation in the most susceptible strain (BALB/c). Incidentally, this previous study had already shown that the effect of priming with MuHV-4 was evident for all doses of PVM, both in terms of survival rate and mean survival time.

To assess pain and distress during the course of infection, animals were assessed twice a day based on a scoring system assigning numerical values to several criteria of animal conditions that were considered signs of morbidity or moribundity, including changes in physical appearance, behavior, temperature, and weight loss. The mice were either euthanized on day-6 p.i. or when they reached one of the three thresholds defined as the end-point. A body weight loss of >20%, a temperature <32 °C, or an illness-score >20 were the cut-off thresholds defined as end-point. The date of death for euthanized mice was marked as the date of euthanasia. None of the animals was found dead during the course of the experiments.

### 2.3. Bacterial Stock Preparation and Use

To assess the functional phenotype of AMs CD11c^+^ cells, a clinical isolate of *Streptococcus pneumoniae* (*S. pneumoniae*) serotype 3 (ATCC 6303; ATCC, Molsheim, France) was prepared. Briefly, the bacterial stock was plated on Columbia blood agar (VWR chemicals, Leuven, Belgium) and incubated overnight. Resultant colonies were cultured at 37 °C in 5% CO_2_ in brain heart infusion broth (BHI) (VWR chemicals, Leuven, Belgium) to exponential phase. Bacteria were then harvested and resuspended in PBS. The infectious dose was verified by plating 10-fold serial dilutions on BHI-agar plates. AMs CD11c^+^ cells were infected with 1 × 10^6^ colony-forming units of *S. pneumoniae* suspended in 100 µL of PBS.

### 2.4. Histological Analysis

The tissues were fixed in 4% formaldehyde, dehydrated in a graded series of ethanol, washed in xylene, and embedded in paraffin wax. Tissue blocks were sectioned at a thickness of 5 μm, and sections were stained with hematoxylin and eosin and evaluated under a light microscope. Lung sections were blindly scored by a board-certified pathologist for several indices of inflammation and cellular infiltrates changes in the conducting airway and alveolar walls. Features considered were the amount of perivascular and peribronchial inflammatory infiltrates, alveolar septal infiltrates and alveolar luminal inflammatory cell exudates, hemorrhage and edema, and degree of epithelial impairment.

### 2.5. Preparation of Cell Suspensions

After euthanasia, the lungs were perfused with 5 mL ice-cold PBS through the right ventricle, then removed and the large airways dissected and discarded. The lungs were removed, and the large airways were dissected from the peripheral lung tissue. The latter was cut into small pieces, transferred into GentleMACS™ C-Tube containing complete HBSS medium, 1 mg/mL collagenase D (Merck Life Science BV, Overijse, Belgium), and 50 μg/mL DNase I (Merck Life Science BV, Overijse, Belgium), and processed with the GentleMACS™ dissociator according to the manufacturer’s instructions (Miltenyi Biotec, Leiden, The Netherlands). Homogenized lungs were passed through a Falcon® 70 μm nylon cell strainer to obtain a single-cell suspension. The latter was centrifuged at 300× *g* for 10 min, and the pellet was resuspended for 5 min into 2 mL of BD Pharm Lyse™ (BD Biosciences, Erembodegem, Belgium) to get rid of the remaining red blood cells. After 2 additional washing steps into PBS/EDTA (10 mM), cells were counted using a Countess™ automated cell counter (Thermo Fisher Scientific Inc., Merelbeke, Belgium), dead cells being excluded based on trypan blue exclusion.

### 2.6. Ex Vivo Culture of AMs CD11c-Positive Cells

AMs CD11c^+^ cells were purified from bronchoalveolar lavage fluids (BALF) single-cell suspensions by positive CD11c MACS selection (Miltenyi Biotech, Leiden, Netherlands) according to the manufacturer’s instructions. Purified cells were then resuspended in complete RPMI 1640 medium (10% FBS, 1 mM l-glutamine) without penicillin/streptomycin (P/S-free media), plated at 1 × 10^5^ cells/well in 24-well plates (500 µL/well) and incubated at 37 °C in a 5% CO_2_ incubator for 1 h. *S. pneumoniae* bacteria were washed twice in PBS, resuspended in P/S-free media at 1 × 10^7^ cfu/mL, and incubated for 30 min at 37 °C. Bacteria were supplemented to cell culture wells with a multiplicity of infection (m.o.i.) of 10. Complete P/S-free media was used as control (basal conditions). Cells were incubated for 1 h at 37 °C, supplemented with 1.5 mL/well complete RPMI 1640 with 1% penicillin/streptomycin, then incubated again for 30 min to get rid of extracellular bacterial cells, washed twice in PBS before measurements. Simultaneously, CD11c^+^ purified cells were infected with PVM at an multiplicity of infection (MOI) of 1. Infected cells were incubated for 2 h at 37 °C in a 5% CO_2_ cell-culture incubator, then washed twice in PBS and brought back at 37 °C before measurements. From this starting point (removal of extracellular bacteria and PVM) onward, supernatants were collected 2, 12, 24, 48, and 72 h after virus/bacteria exposure for cyto/chemokine assays.

### 2.7. Flow Cytometry

Living cells were resuspended in PBS supplemented with 0.5% of BSA and 0.1% of sodium azide. Cells were first incubated on ice for 10 min with purified rat anti-mouse CD16/CD32 antibody (BD Bioscience, Erembodegem, Belgium) before being marked 30 min on ice with various panels of fluorochrome-conjugated antibodies: CD45 (30-F11, FITC), CD11b (M1/70, eFluor 450), CD24 (M1/69, APC), CD117 (2B8, APC) from eBioscience (Thermo Fisher Scientific Inc., Merelbeke, Belgium); CD3ε (145-2C11, Horizon PE-CF594), CD4 (GK 1.5, PE-Cy7), CD8α (53-6.7, APC-H7), CD11c (HL3, PE-Cy7), CD19 (1D3, PerCP-Cy5.5), CD49b (DX5, BV421), IgE (R35-72, FITC), Ly6C (AL-21, BV605), Ly6G (1A8, Alexa Fluor 700), MHC II (M5/114.15.2, Horizon V500), NKp46 (29A1.4, Horizon V450), SiglecF (E50-2440, Horizon PE-CF594), TCRγδ (GL3, BV711) from BD Biosciences; CD69 (H1.2F3, APC), CD64 (X54-5/7.1, PE), CD103 (2E7, PerCP-Cy5.5) from Biolegend (Amsterdam, Netherlands); mCD1d PBS-57 tetramer (PE) from the NIH Tetramer Core Facility (Emory University, Atlanta, GA), and mPDCA-1 (JF05-1C2.4.1, APC) from Miltenyi Biotec. To assess the absolute cell number for each cell subpopulations and control the between-mice experiment’s reproducibility, CountBright™ Absolute Counting Beads (Thermo Fisher Scientific Inc., Merelbeke, Belgium) were added to the marked cells. Data were acquired on BD FACS Aria III, BD LSRFortessa, and BD FACS Verse flow cytometers using BD FACSDiva software (BD Biosciences). Cell viability was assessed by propidium iodide viability assay (data not shown). Compensation and data analysis were performed offline using FlowJo software 10.7.0 (TreeStar, Ashland, OR, USA). Cytospins were prepared from sorted cells performed on FACSAria III instrument and stained with May–Grünwald Giemsa (Merck Life Science BV, Overijse, Belgium) before microscopy analysis. The lymphoid and myeloid lungs subpopulations were identified using three distinct gating strategies, followed by a series of cytospins to provide visual control of the gated cell populations (gating strategy available in Figures S1–S3). Fluorescence-minus-one (FMO) controls were used to set flow cytometric gates (Figures S4 and S5).

### 2.8. Reverse Transcription and Real-Time PCR Analysis

In order to quantify the PVM virus genomic RNA and to demonstrate a possible reactivation of the MuHV-4 virus in the lungs after the PVM infection, a section of the *sh* gene (PVM) and of the *DNA polymerase* (ORF9) transcript (MuHV-4) were quantified by dual-standard curve reverse transcription real-time PCR. Lung total RNA was extracted and purified using the RNeasy Mini Kit (Qiagen, Antwerp, Belgium). Following TURBO DNase treatment (Thermo Fisher Scientific Inc., Merelbeke, Belgium), 1 μg of total RNA samples were reverse transcribed using random hexamers and TaqMan reverse transcription reagents (Thermo Fisher Scientific Inc., Merelbeke, Belgium), a no reverse transcriptase control being included. RT-PCR products were run in triplicate, with ABI TaqMan Universal Master Mix II, with UNG, primer-probe mixes for the PVM *sh* gene (Forward: 5′-GCC GTC ATC AAC ACA GTG TGT-3′; Reverse: 5′-GCC TGA TGT AGC AAT GCT T-3′; Probe: 5′-CGC TGA TAA TGG CCT GCA GCA-3′), the MuHV-4 *DNA polymerase* (Forward: 5′-ACA GCA GCT GGC CAT AAA GG-3′; Reverse: 5′-TCC TGC CCT GGA AAG TGA TG-3′; Probe: 5′-CCT CTG GAA TGT TGC CTT GCC TCC A), and the *Mus musculus* tyrosine 3-monooxygenase/tryptophan 5-monooxygenase activation protein, zeta polypeptide (*ywhaz*) (Forward: 5′-TGC AAC GAT GTA CTG TCT CTT TTG-3′; Reverse: 5′-CGG TAG TAG TCA CCC TTC ATT TTC A-3′; Probe: 5′-TCC CCA ATG CTT CGC AAC CAG AAA GCA-3′), and cDNA or standard in a 20 µL final volume (Applied Biosystems). Thermal cycling parameters for the ABI StepOnePlus absolute quantitation program included 50 °C for 2 min (UNG incubation), 95 °C for 10 min (AmpliTaq Gold activation), and 40 amplification cycles alternating 95 °C for 15 s and 55 °C for 1 min. Standard curves included serial dilutions (10^9^, 10^8^, 10^7^, 10^6^, and 10^5^ molecules/reaction) of *ywhaz* (NM_011740.3) and *DNA polymerase* (NC_001826.2) coding sequences and serial dilutions (10^10^, 10^9^, 10^8^, 10^7^, 10^6^, and 10^5^ molecules/reaction) of the full-length PVM *sh* gene (NC_006579.1). Experimental triplicate data points were interpolated to linear standard curves over the concentration ranges indicated. Samples calculations from data generated by this method were performed as described [18]. Real-time PCR results were confirmed by median tissue culture infectious dose (TCID_50_) titration of whole-lung homogenates.

### 2.9. Cytokines and Chemokines Assays

Protein levels of IL-1β, IL-2, IL-4, IL-5, IL-6, IL-10, IL-12p70, IL-13, TNF-α, IFNγ, monocyte chemoattractant protein-1 (MCP-1, CCL2), macrophage inflammatory protein-1α (MIP-1α, CCL3), macrophage inflammatory protein-1β (MIP-1β, CCL4), RANTES (CCL5), eotaxin (CCL11), neutrophil-activating protein-3 (NAP-3, CXCL1), macrophage inflammatory protein 2 (MIP-2, CXCL2), IFN-inducible protein-10 (IP-10, CXCL10), granulocyte-macrophage colony-stimulating factor (GM-CSF), granulocyte colony-stimulating factor (G-CSF), vascular endothelial growth factor (VEGF) and granzyme B were measured in 75 μL samples from each lung homogenate using a Mouse Premixed Multi-Analyte kit (Magnetic Luminex assay; cat LXSAMSM; R&D Systems) according to the manufacturer’s instructions. The samples were analyzed on the Luminex 100/200 cytometer using xPONENT^®^ 4.5 software. Results, generated using lung tissue homogenates, were normalized to total protein per sample, determined via BCA protein assay (Pierce) with BSA standards.

### 2.10. Statistical Analysis

Kaplan–Meier survival curves were compared using the log-rank test. Differences between groups were assessed with Student’s *t*-test. Multiple-group comparisons were carried out by one-way analysis of variance followed by the Newman–Keuls test. Kaplan–Meier survival curves were compared using the log-rank test. *p*-values < 0.05 were considered significant at a 95% confidence interval for all analyses.

## 3. Results

### 3.1. Genetic Background of MuHV-4 Primed Mice Has a Significate Impact on the Survival Outcome of a Lethal Pneumovirus Infection

**Figure 1:**
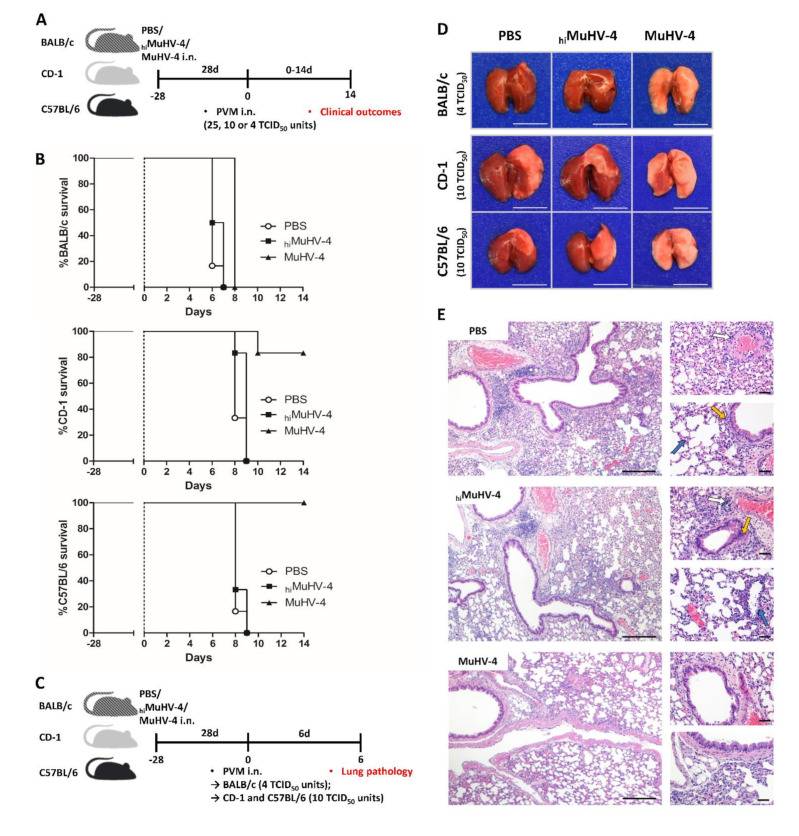
Past MuHV-4 infection provides protection against subsequent pneumoviral disease. (**A**) Schematic of the survival study. On day -28, CD-1, BALB/c, and C57BL/6 mice were inoculated intranasally with 1.4 × 10^4^ TCID_50_ units of MuHV-4, _hi_MuHV-4, or PBS. On day 0, mice were intranasally inoculated with 4, 10, or 25 TCID_50_ units of PVM, and the survivability of each mice strain was monitored. (**B**) Survival study enrolling each mice strains given either MuHV-4, _hi_MuHV-4, or PBS intranasally 28 days prior to 4 TCID_50_ units of PVM infection (*n* = 6 mice/mouse strain/priming condition; representative of three independent experiments). (**C**) Schematic of experimental protocol. On day −28, CD-1, BALB/c, and C57BL/6 mice were inoculated intranasally with 1.4 × 10^4^ TCID_50_ units of MuHV-4, _hi_MuHV-4, or PBS. On day 0, mice were intranasally inoculated with PVM (4 TCID_50_ units for BALB/c mice and 10 TCID_50_ units for CD-1 and C57BL/6 mice). Finally, on day 6, lungs were removed for further examinations. (**D**) Macroscopic aspect of lungs excised from each mouse strain on day 6 after PVM infection (scale bar = 1 cm). (**E**) Representative sections of BALB/c mice H&E-stained lung tissues on day 6 after PVM infection (4 TCID_50_ units) (histopathological reactions are similar among each mice strain). Conspicuous alveolar (blue arrows), peribronchiolar (yellow arrows), and perivascular (white arrows) inflammatory infiltrations were constant among PBS- and _hi_MuHV-4-primed mice, while occasional in MuHV-4-primed mice. Scale bar = 200 µm for left panels, 50 µm for right panels.

To determine the resistance of each primed mouse strain and to address if the priming of the respiratory mucosa with living MuHV-4 would modify the outcome of a lethal pneumovirus infection, BALB/C, CD-1, and C57BL/6 mice were primed by living MuHV-4, Heat-inactivated MuHV-4 (_hi_MuHV-4), or PBS inoculation and subjected 28 days later (day 0) to different infectious doses of PVM (25, 10 or 4 TCID_50_ units). Following the infection, a longitudinal study was applied with clinical scoring, subcutaneous temperature, and body weight values measurement over the next 14 days (Figure 1A).

MuHV-4 primed mice presented a higher median survival time in each mouse strain following 25, 10, or 4 TCID_50_ units PVM inoculation (Figure S6). No mice survived following 25 TCID_50_ units of PVM inoculation. However, following 10 TCID_50_ units of PVM inoculation, the CD-1 and C57BL/6 mice presented their first cases of survival following MuHV-4 priming while all the BALB/c mice succumbed to PVM infection. Finally, 4 TCID_50_ units of PVM inoculation caused lethal disease in 100% of the PBS mock-primed mice, with mean survival times of 6.2, 8.2, and 8.3 days in BALB/c, CD-1, and C57BL/6, respectively (Figure 1B). In BALB/c, subcutaneous temperature decreased slowly for 4 days and then rapidly to the end-point. In CD-1 and C57BL/6, it remained stable for 5 days and then abruptly reached the end-point within 72 h. At the cut-off point, live weight loss peaked at ~15% in BALB/c and ~20% in CD-1 and C57BL/6 (Figure S7A,B).

Similarly, _hi_MuHV-4 inoculation 28 days before PVM infection resulted in comparable clinical manifestations as in the PBS-primed group (Figure 1B, Figures S6 and S7), excluding therefore the hypothesis of a protective response developed against the sole antiviral charge of the MuHV-4.

Conversely, prior infection with living MuHV-4 significantly improved the PVM-associated clinical picture. Survival rate increased from 84% to 100% in CD-1 and C57BL/6, respectively (Figure 1B). The disease remained, however, consistently fatal in BALB/c, but the mean survival time increased from 6.2 to 8.0 days (*p* < 0.01). The course of the temperature drop was delayed. The nadir was reached later and, with the exception of BALB/c, was never less than about 33 °C. Normal temperature recovered rapidly in CD-1 (within 4 days), much more slowly in C57BL/6. The extent of weight loss was unaffected by living MuHV-4 infection. Initial body weight recovered quickly in CD-1 and slowly in C57BL/6 (Figure S7A,B). These results highlight an influence of the genetic background on the survivability in front of a lethal pneumovirus inoculation.

For further investigations, as the peak of induced disease is reached 6 days after PVM inoculation [26], lungs modifications were analyzed on day 6 post infection (Figure 1C). Additionally, based on our results from the survival curves (Figure S6), the infectious dose for PVM inoculations were defined as 4 TCID_50_ units in BALB/c mice to maintain a 100% survival state at the time of measurement and 10 TCID_50_ units in CD-1 and C57BL/6 mice to ensure significant immune reaction. The differences in mortality rate, weight, and temperature loss between primed (MuHV-4) and mock-primed (PBS and _hi_MuHV-4) mice correlated with the pathological findings made on lungs collected on day 6 post infection (p.i.) (Figure 1D,E). Macroscopic examination of mock-primed (PBS and _hi_MuHV-4) mice showed similar lungs alterations 6 days following PVM inoculation (Figure 1D): expanded, red-colored tissue, rounded edges, gelatinous consistency, prominent edema, and acute inflammation. A significant lung weight gain was measured compared to the lungs of age-, sex-, and strain-matched uninfected mice, of the order of +50% in BALB/c and +90% in C57BL/6 and CD-1 (Figure S7C). Conversely, MuHV-4 primed mice presented a reduced lung weight increase by about 25% in all three strains, and macroscopic examination revealed how a past infection with live MuHV-4 reduces the lung inflammation associated with a subsequent pneumoviral infection. The volume and consistency of the lungs remained unchanged, and areas of congestion were rare (Figure 1D). Finally, histopathological examination of the mock-primed, PVM-infected mice demonstrated alterations typical of a prominent, diffuse alveolar and interstitial edema and diffuse alveolitis with diffuse recruitment of granulocytes and conspicuous perivascular/peribronchial cuffing with monocytic leucocytes (Figure 1E). On the other hand, microscopical inspection of MuHV-4 primed mice showed a moderate excess of mononuclear cells accumulated in the interalveolar septa and forming cuffs around the bronchioles and vessels was noticed with little edema and alveolitis and no diffuse recruitment of granulocytes.

### 3.2. MuHV-4 Priming Affects the Leukocyte Subpopulations Recruitment in Lungs Prior and After PVM Inoculation in a Murine Strain-Dependent Manner

**Figure 2:**
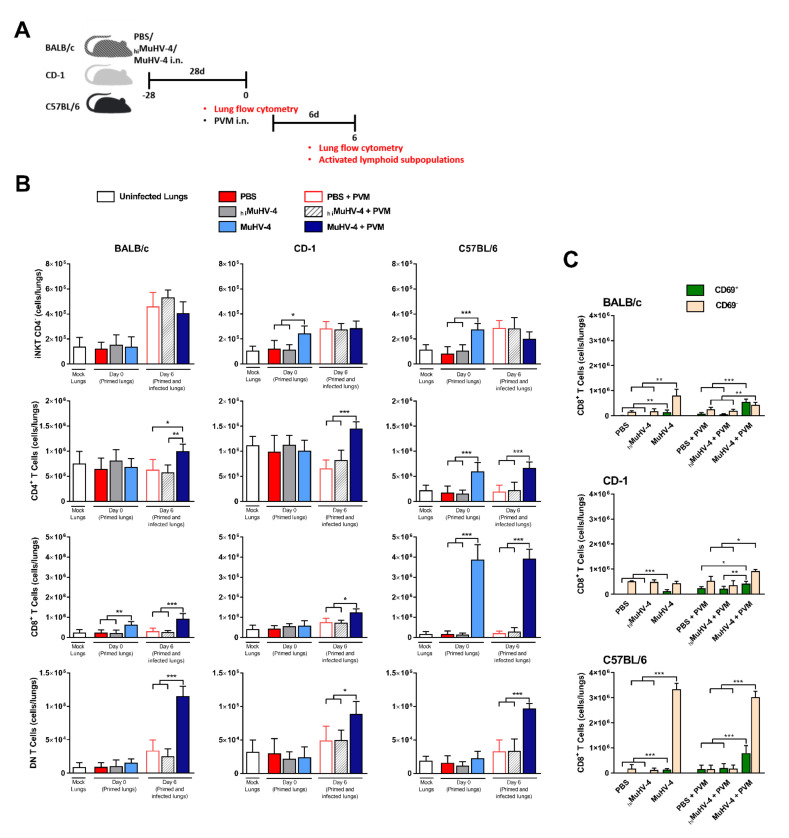
MuHV-4 priming adjusts lung lymphoid subpopulations both before and 6 days after exposure to PVM. (**A**) Schematic of experimental protocol. On day −28, CD-1, BALB/c, and C57BL/6 mice were inoculated intranasally (i.n.) with 1.4 × 10^4^ TCID_50_ units of MuHV-4, _hi_MuHV-4, or PBS. Each cohort was then divided into two sub-cohorts based on whether euthanasia was performed just before (day 0) or 6 days after intranasal inoculation of PVM. On day 0, the lungs of the first half of the mice were removed for flow cytometry analysis, while the other half of the mice were infected with PVM. On day 6, the lungs were removed and analyzed by flow cytometry. (**B**) Enumeration of absolute lung lymphoid subpopulations in mock-primed (_hi_MuHV-4 and PBS) and MuHV-4-primed mice on days 0 (primed lungs) and 6 (primed and infected lungs) following PVM inoculation. (**C**) Absolute number of activated (CD69^+^) and inactivated (CD69^−^) lungs CD8^+^ T-cell subsets in mock- or MuHV-4 primed mice measured on days 0 and 6 following PVM inoculation (mean ± SD, with *n* = 6 mice/mouse strain/priming condition; representative of three independent experiments). Mock lungs, primed lungs (day 0), and primed/infected lungs (day 6) are considered separately from each other for statistical analysis. Significantly different means are highlighted, with * *p* < 0.05, ** *p* < 0.01, or *** *p* < 0.001. n.s., not significant (one-way ANOVA and Bonferroni post-tests).

**Figure 3:**
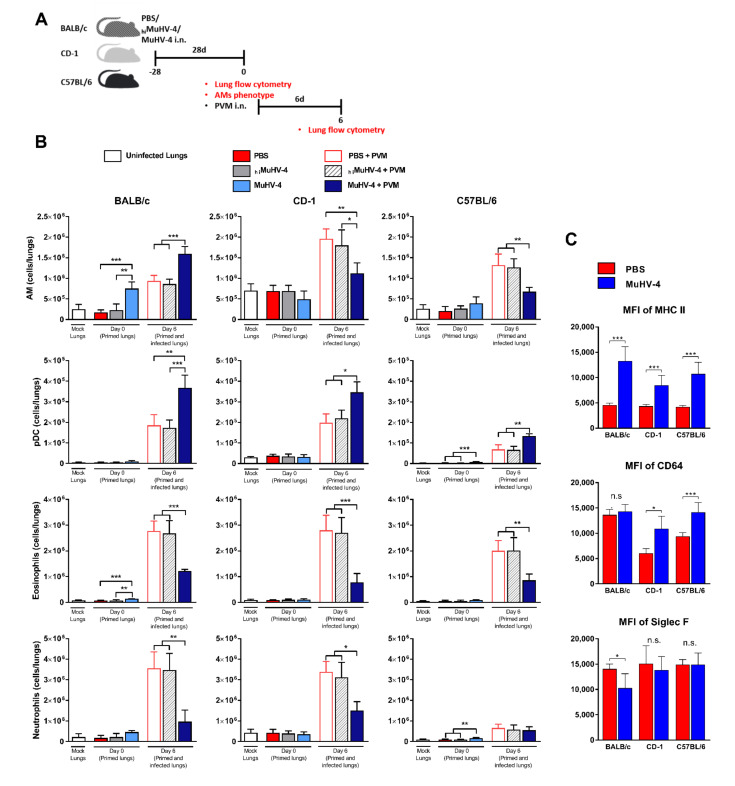
MuHV-4 priming adjusts lung myeloid subpopulations both before and 6 days after exposure to PVM. (**A**) Schematic of experimental protocol. On day −28, CD-1, BALB/c, and C57BL/6 mice were inoculated intranasally (i.n.) with MuHV-4, _hi_MuHV-4, or PBS. Each cohort was then divided into two sub-cohorts based on whether euthanasia was performed just before (day 0) or 6 days after intranasal inoculation of PVM. On day 0, the lungs of the first half of the mice were removed for flow cytometry analysis, while the other half of the mice were infected with PVM. On day 6, the lungs of the remaining mice were removed and analyzed by flow cytometry. (**B**) Enumeration of absolute lung myeloid subpopulations in mock-primed (_hi_MuHV-4 and PBS) and MuHV-4-primed mice on days 0 (primed lungs) and 6 (primed and infected lungs) following PVM inoculation. (**C**) Mean fluorescence intensity (MFI) of MHC II, CD64, and SiglecF expression on AMs 28 days after priming either with MuHV-4 or PBS (mean ± SD, with *n* = 6 mice/mouse strain/priming condition; representative of three independent experiments). Mock lungs, primed lungs (day 0), and primed/infected lungs (day 6) are considered separately from each other for statistical analysis. Significantly different means are highlighted, with * *p* < 0.05, ** *p* < 0.01, or *** *p* < 0.001. n.s., not significant (one-way ANOVA and Bonferroni post-tests).

To highlight the pulmonary cellular modifications following MuHV-4 inoculation and to address the influence of the genetic background on the immune response development against a heterologous PVM infection, the different leukocyte subpopulations of each mice strain were analyzed successively in naïve mice, in mice mock- or MuHV-4 primed for 28 days, and in mock- or MuHV-4 primed mice infected by PVM for 6 days (Figure 2A and Figure 3A). A recap table for changes observed in the leukocyte’s subpopulations in the three different strains compared to naive mice can be found in Supplementary Table S1.

#### 3.2.1. Whole-Lung Cellular Environment in Each Mice Strain Native State

In the first step of our experiment, the pulmonary cellular environment of each mice strain was assessed in its native state. In naïve mice, normalized enumeration of leucocyte subsets revealed that lungs of CD-1 mice are constitutively infiltrated by higher numbers of γδT cells, AMs, Ly6C^low^ monocytes (Ly6C^lo^ Mo), Ly6C^high^ monocytes (Ly6C^hi^ Mo), CD11b^+^ dendritic cells (CD11b^+^ DCs), plasmacytoid dendritic cells (pDCs), mast cells, neutrophilic and basophilic granulocytes. Moreover, lungs of BALB/c contain 2.5-fold more NK cells, C57BL/6 about four times less CD4^+^ T and B cells and CD-1 far less non-alveolar macrophages (nAMs) (CD11b^+^CD11c^+^CD64^int^SiglecF^−^) (Material not intended for publication: G. Gilliaux, D. Desmecht, University of Liège, Liège, Belgium, 2019).

#### 3.2.2. Whole-Lung Cellular Modifications 28 Days after Mock or Muhv-4 Inoculation

In a second step, the pulmonary cellular modifications following mock or MuHV-4 nasal inoculation (day 0 of PVM infection) were analyzed in each mouse strain.

- Lymphoid lineage; 28 days after intranasal injection of PBS or _hi_MuHV-4, the pulmonary lymphoid subpopulations remained unchanged (*p* > 0.05) (Figure 2B). Further, the injection of _hi_MuHV-4 did not increase the proportion of activated lymphoid cells (CD69^+^) compared to the saline injection (data not shown). Conversely, after inoculation of MuHV-4, the overall lung leukocyte counts increased, as did the count of some subpopulations, albeit heterogeneously between strains (*p* < 0.05): iNKT/CD4^−^ cells (in CD-1 and C57BL/6 strains), NK cells (C57BL/6), B cells (C57BL/6), CD4^+^ T cells (C57BL/6), CD8^+^ T cells (BALB/c and, most importantly, C57BL/6 with a 20-fold increase) and γδT cells (BALB/c) (Figure 2B and Figure S8). Further, the general rule, valid for all three mouse strains, was that past infection with MuHV-4 virus strongly increased the absolute number of activated (CD69^+^) CD8^+^ cells (Figure 2C);
- Myeloid lineage; similarly, myeloid subpopulations were not altered after intranasal injection of saline or _hi_MuHV-4. On the contrary, prior injection of MuHV-4 resulted in strain-specific expansions of specific subpopulations: AMs and eosinophilic granulocytes in BALB/c, pDCs, mast cells, and neutrophilic granulocytes in C57BL/6 and nAMs (5-fold) in CD-1 (Figure 3B, Figures S9 and S10). Further, the comprehensive gating strategy used to distinguish bona fide AMs (CD11b^−^CD11c^hi^CD64^hi^SiglecF^hi^) from other myeloid cells revealed both shared and specific phenotypic changes. Most importantly, in all three strains, AMs examined four weeks past MuHV-4 infection displayed a significantly increased MHC II expression compared to PBS-treated mice (Figure 3C). Strain-specific changes consisted of a decreased (BALB/c) or increased (C57BL/6 and CD-1) expression of SiglecF and CD64 expression, respectively. Expression of other AM surface markers CD11b, CD11c, and Ly6C remained unchanged.

#### 3.2.3. Whole-Lung Cellular Modifications 6 Days Post PVM Inoculation

In the final step of our experiment, the analysis on day 6 post PVM inoculation in mock or MuHV-4-primed mice highlighted significant leukocyte proportion modifications. Various immune reactions were measured according to the mice strain considered following PVM infection, and this, independently of the influence of the MuHV-4-priming on the immune system. Thus, the enumerated leukocyte subsets are essential for different immunological tasks, and therefore it is theoretically possible that the observed constitutive strain heterogeneity influences respiratory immunity to different pathogens.

- Lymphoid lineage; the recruitment pattern of lymphoid subpopulations in mock-primed mice was similar in all three strains, with an increase in the numbers of iNKT CD4^−^, iNKT CD4^+^, and NK cells (more marked in BALB/c and CD-1 than in C57BL/6) while the numbers of B cells, CD4^+^, CD8^+^, γδT, and DN T cells remained stable (Figure 2B and Figure S8). Qualitatively speaking, the PVM caused an increase in the proportion and absolute number of CD69^+^, thus activating lymphoid cells. However, past infection with MuHV-4 further modified the leukocyte response to PVM infection. Compared to mock-primed mice, an expansion of the pool of CD4^+^, CD8^+^, and DN T cells was observed in all three strains (Figure 2B), along with an exacerbation of the expansion of the iNKT CD4^+^ cell subpopulation in C57BL/6 and strain-specific expansions of specific subpopulations already measured on day 0 following MuHV-4-priming: B cells in C57BL/6 and γδT cells in BALB/c mice (Figure S8). Qualitatively speaking, the general rule that emerged following MuHV-4 priming was an increase in the absolute number of activated CD8^+^ T cells 6 days post PVM inoculation (Figure 2C);
- Myeloid lineage; all mock-primed mice infected by the PVM showed an expansion of the AMs, pDCs, neutrophilic and eosinophilic granulocyte populations (Figure 3B). Some changes were strain-specific: In CD-1, there was (i) a marked expansion of the Ly6C**^high^** Mo pool without any change in the Ly6C**^low^** Mo pool, whereas the opposite was true in BALB/c and C57BL/6 and (ii) a marked expansion of the nAMs and CD103^+^ dendritic cells (CD103^+^ DCs) pool (Figures S9 and S10). Finally, MuHV-4-priming induced significant modifications in myeloid cells. Two effects of prior infection with MuHV-4 on PVM-dependent reactions were shared by all three mouse strains compared to mock-primed mice: enhancement of the pDC pool expansion and, on the contrary, blunting of neutrophilic and eosinophilic granulocyte expansions (Figure 3B). Further, some changes induced by the MuHV-4 priming were strain-specific: (i) expansion of the AMs and mast cells pools were exacerbated in BALB/c while it was blunted in the other two strains (Figure 3B) and (ii) enhanced expansion of nAMs and diminished expansion of Ly6C^hi^ Mo and CD103^+^ DCs subpopulations in CD-1 mice (Figures S9 and S10). Two differences deserve specific attention. We found a striking elevation of lung pDCs in CD-1 mice in comparison to inbred strains. As lung pDCs represent a key DC subset responding to viral infections, higher pDC numbers may influence the pathogenesis of respiratory viral infection. Further, lung CD103^+^ DCs were far more numerous in CD-1 mice than in other mice. Given the critical role of respiratory CD103^+^ DCs for activation of naive CD8^+^ T cells in respiratory viral infections [27,28], an expanded pool of CD103^+^ DCs may impact the strain-dependent capacity to generate viral CD8^+^ T-cell immunity.

### 3.3. Muhv-4 Priming Impact The PVM Recovery in A Murine Strain-Dependent Manner and Reduce PVM-Induced Lung Pro-Inflammatory Cyto/Chemokine Contents

**Figure 4:**
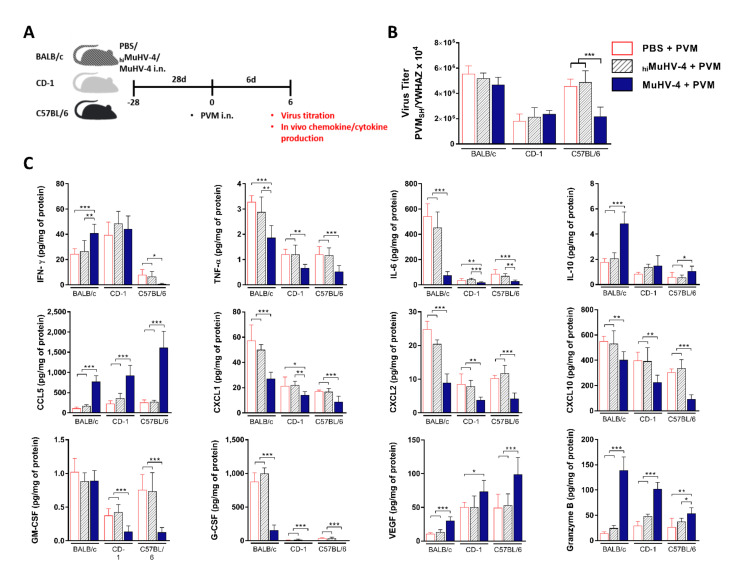
MuHV-4 priming results in strain-dependent virus recovery modification and reduced lung pro-inflammatory cyto- and chemokine contents during pneumoviral disease. (**A**) Schematic of experimental protocol. On day −28, CD-1, BALB/c, and C57BL/6 mice were inoculated intranasally with MuHV-4, _hi_MuHV-4, or PBS. On day 6 after PVM inoculation, lungs were prepared for virus titration and chemokine/cytokine assays. (**B**) PVM titer determined by quantitative RT-qPCR applied on whole-lung homogenate. Results are expressed as PVM *sh* copies per 10^4^ *ywhaz* copies. (**C**) Lung cyto- and chemokine concentrations normalized to sample total protein content. All results are reported as mean ± SD, with *n* = 6 mice/mouse strain/priming condition, representative of three independent experiments. Significantly different means are highlighted, with * *p* < 0.05, ** *p* < 0.01, or *** *p* < 0.001. n.s., not significant (one-way ANOVA and Bonferroni post-tests).

To determine the effect of the MuHV-4 priming on the PVM recovery, the virus titer was determined on mock and MuHV-4-primed mice 6 days post PVM inoculation in each mice strain. As the virus recovery alone cannot predict disease outcome or long-term survival and as the morbidity and mortality in response to severe respiratory virus infection can result in large part from uncontrolled amplification of pro-inflammatory signaling networks, the cytokine and chemokine production was also determined on the same day (Figure 4A).

In lung extracts from mock-primed mice, viral RNA copy numbers reached high levels, with slightly, albeit significantly lower counts in CD1 mice (*p* < 0,05). On the other hand, PVM titers obtained from MuHV-4-primed lung extracts were not modified in CD1 and BALB/c mice. Conversely, they were significantly decreased in C57BL/6 (~3-fold) (Figure 4B). Importantly, no sign of MuHV-4 reactivation was noted in primed lungs following infection with PVM. Indeed, qPCR titrations applied, 6 days post PVM infection, on MuHV-4 gene *DNA polymerase* showed no results (Material not intended for publication: G. Gilliaux, D. Desmecht, University of Liège, Liège, Belgium, 2020). On the other hand, chemokine/cytokine assay applied of MuHV-4-primed mice demonstrated a systematic suppression of several cytokines and chemokines associated with the PVM inflammatory pathology independently of the genetic background: TNF-α, IL-6, chemokines neutrophil-activating protein-3 (CXCL1), macrophage inflammatory protein 2 (CXCL2), IFN-inducible protein-10 (CXCL10) and the granulocyte colony-stimulating factor (G-CSF) were decreased significantly (Figure 4C). Conversely, the concentrations of the chemokine CCL5, the vascular endothelial growth factor (VEGF), and granzyme B were increased in each mice strain. Finally, concentrations of IL-1β, IL-5, IL-12p70, IL-13, and CCL11 presented no significant differences (Figure S11). In a second time, strain-specific effects were also highlighted: lung IFNγ content was unchanged in CD-1, increased in BALB/c, and decreased in C57BL/6. IL-10 content was similar in CD-1, while it was increased in both inbred strains (Figure 4C). Finally, a pattern specific to BALB/c was detected: PVM-induced secretion of CCL3, CCL4, and IL-4 was enhanced while it remained similar in CD-1 and C57BL/6. For CCL2, it was the opposite: decrease in BALB/c and stability in the other two strains. Granulocyte-macrophage colony-stimulating factor (GM-CSF) did not vary while its concentration was decreased in CD-1 and C57BL/6 (Figure S11).

### 3.4. Past Muhv-4 Infection Alters Ams Phenotype Independently of Circulating Monocytes

**Figure 5:**
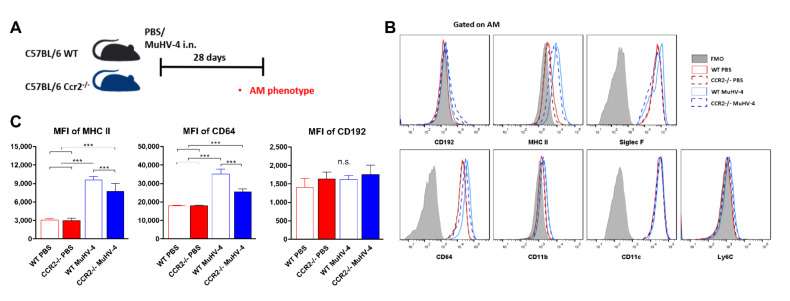
MuHV-4 priming educates AMs without help from monocyte-derived macrophages. (**A**) Schematic of experimental protocol. Wildtype (WT) and CCR2^−/−^ C57BL/6 mice were inoculated intranasally (i.n.) with either MuHV-4 or PBS, and their lungs were analyzed 28 days later. (**B**) Flow cytometry plot showing expression of AMs’ surface markers 28 days post priming. (**C**) Magnitude of several markers expression at the AM’s surface, as determined by mean fluorescence intensity (MFI) 28 days post priming. All results are reported as mean ± SD, with *n* = 6 mice/mouse strain/priming condition, representative of three independent experiments. Significantly different means are highlighted, with *** *p* < 0.001. n.s., not significant (one-way ANOVA and Bonferroni post-tests).

In previous experiments, we have shown that exacerbation of MHC II expression on the surface of lung macrophages is one of the key events caused by MuHV-4 priming (Figure 3C). This phenomenon is described in the literature as bone-marrow-derived monocytes (Ly6G^−^, SiglecF^−^, CD11b^+^, Ly6C^hi^, CCR2^+^) colonizing the empty alveolar niche formed by the MuHV-4 infection and differentiating into AMs that are almost identical transcriptionally to lung-resident AMs and express higher levels of MHC II molecules, and reduced level of SiglecF [15]. However, evidence also suggests AMs to be maintained independently of monocytes under inflammatory conditions [29,30,31]. In order to dissect the ontogeny of this alteration, we examined the AMs phenotype of 28 days naïve or MuHV-4 primed C57BL/6 wildtype (WT) mice and CCR2-deficient (CCR2^−/−^) mice lacking classical Ly6C^hi^ monocytes, cells suspected to replenish tissue macrophage niches (Figure 5A). We first confirmed the absence of Ly6C^hi^ monocyte recruitment to the lung monocyte population in CCR2^−/−^ mice (Figure S12). Secondly, we showed that the expression level of MHC II and CD64 in lung macrophages is increased after priming with MuHV-4 in both WT and CCR2^−/−^ mice (Figure 5B), although significantly higher in WT mice (Figure 5C). CCR2-specific CD192 antibody levels remained, however, unchanged in AMs both in naïve or MuHV-4 primed mice (Figure 5B,C). Other AMs’ surface markers (CD11b, CD11c, and Ly6C) remained unchanged. As the AMs phenotype modification emerges in both WT and CCR2^−/−^ mice, these data disprove the hypothesis of a monocytic origin in the modification of the phenotype of AMs after MuHV-4 priming in C57BL/6 mice.

### 3.5. MuHV-4 Primed AMs Require Recruited Monocytes to Exert Antibacterial and Reduced Inflammatory Phenotypes

**Figure 6:**
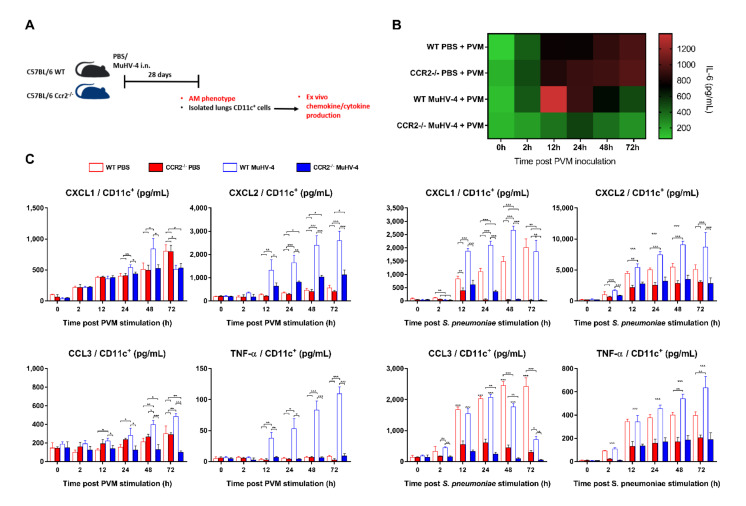
MuHV-4 priming resets AMs’ immediate response to PVM and *Streptococcus pneumoniae*. (**A**) Schematic of experimental protocol. WT and CCR2^−/−^ C57BL/6 mice were inoculated intranasally (i.n.) with either MuHV-4 or PBS; lung CD11c^+^ AMs were then isolated 28 days after and ex vivo stimulated by PVM or *S. pneumoniae* inoculation before cyto- and chemokines measurement over 72 h. (**B**) Heatmap of IL-6 production in CD11c^+^ AMs harvested from 28 days PBS or MuHV-4 primed mice, at 0, 2, 12, 24, 48, and 72 h post PVM inoculation. (**C**) Supernatant cyto- and chemokine concentrations normalized to sample total protein content. All results are reported as mean ± SD, with *n* = 6 mice/mouse strain/priming condition, representative of three independent experiments. Significantly different means are highlighted, with * *p* < 0.05, ** *p* < 0.01, or *** *p* < 0.001. n.s., not significant (one-way ANOVA and Bonferroni post-tests).

One hallmark of innate immune memory is its ability, via innate immune cells stimulation, to enhance non-specific innate protection against heterologous microbial infection [18,32]. Therefore, To address the relationship between primed MHC II^hi^ AMs, monocyte recruitment, and protective innate immunity in front of viral or bacterial pathogens, we purified CD11c^+^ cells from bronchoalveolar lavage fluids (BALF) of mock or MuHV-4-primed WT or CCR2^−/−^ C57BL/6 mice, and we examined whether the lung primed AMs had similar cyto-/chemokines production in response to a viral (PVM) or bacterial challenge by Streptococcus pneumoniae, a common respiratory bacterial pathogen [33] (Figure 6A). Simultaneously, AMs activated by prior adenoviral infection in vivo were shown to produce significantly more CXCL1 and CXCL2 when exposed to S. pneumoniae [29]. These observations prompted us to examine whether priming by MuHV-4 would also reset the response pattern of AMs upon exposure to pathogens. As the regulatory monocytes in BALF responsible for the AMs replacement are defined as CD11c^−^, CD19^−^Ly6G^−^SiglecF^−^CD11b^+^CCR2^+^Ly6C^hi^ cells [15], our CD11c^+^ isolation ensured that only resident AMs were considered in this study. Upon PVM inoculation, a significant increase in IL-6 production was detected in mock-primed WT and CCR2^−/−^ mice, starting at 12 h post infection and continuously increasing to the last measure applied 72 h post inoculation (Figure 6B). Conversely, MuHV-4 primed CD11c^+^ cells from WT mice developed a stronger but much shorter increase in IL-6 secretion (Figure 6B), exacerbation of CCL3 secretion, and the elicitation of new responses (CXCL2 and TNF-α) upon exposure to PVM (Figure 6C). In all cases, this specific response of the MuHV-4 primed cells was weakened or abrogated if the cells were derived from CCR2^−/−^ mice. On the other hand, MuHV-4-primed cells exposed to Streptococcus pneumoniae did not induce any IL-6 production but triggered an exacerbation of CXCL1, CXCL2, and TNF-α secretion and attenuation of CCL3 secretion. Again, these effects of MuHV-4 priming were absent in CCR2^−/−^ mice. Taken together, these results suggest that priming with MuHV-4 alters the response of resident AMs to PVM and Streptococcus pneumoniae via an enhanced inflammatory response limited in time and via accelerated chemokine and cytokine responses. Moreover, even if the AMs phenotype modification following MuHV-4 priming does not seem to come from monocytes origin (Figure 5), these latter seem necessary for the AMs alteration of their cyto/chemokine response.

## 4. Discussion

In this study, we demonstrated that prior MuHV-4 infection attenuates host response to subsequent pneumoviral disease. Indeed, the disease characteristics associated with PVM infection without priming confirmed the clinical findings, nature of macro- and microscopic lesions, and viral loads previously reported [20,26,34]. However, in the MuHV-4-primed group, PVM-infected mice survival rate or resistance was significantly increased. Further, macroscopic lesions observed six days after PVM infection were drastically reduced, and the inflammation-dependent weight gain of the lungs was highly attenuated. Histologically, the most remarkable difference between mock- and MuHV-4-primed mice was the significant decrease in the recruitment of eosinophilic and neutrophilic granulocytes. Environmental effects are unlikely to account for the differences detected since specific pathogen-free, age-matched animals were received from the same provider and housed under specific pathogen-free conditions. Since MuHV-4-priming did not cause a biologically relevant reduction in lung viral loads (with the exception of C57BL/6 mice) but increased resistance/survivability of the mice, it is inferred that imprinting primarily affects the host response to subsequent exposure to pneumovirus. To isolate the MuHV-4 effect independently of the genetic background effect, we focused on the responses to MuHV-4 infection that are shared by the three strains, among which lymphoid/myeloid improvement taking place before and cytokine/chemokine repositioning occurring during PVM infection. The general rule, valid for all three mouse strains, was that past infection with MuHV-4 virus increased significantly the absolute number of activated lungs CD8^+^ cells on day 28 p.i. (Figure 2C), thus before PVM infection. Activation and proliferation of antigen-independent bystander CD8^+^ T cells and their function during subsequent infections have remained controversial. Though some studies have assigned them a role during viral infections [35,36], other studies suggested that bystander activation of CD8^+^ T cells is insignificant in terms of functional roles [37,38,39,40]. Recently, however, studies have demonstrated that the host priming by a pathogen induced a long-term infiltration of CD8^+^ T cells in the lungs. These latter are capable of expressing protective effector functions in response to heterologous challenge, and that could act as “helper” cells, via IFNγ production, to support dendritic cells production of cytokines such as IL-12p70 [41,42]. Similarly, it has been shown that CD8^+^ lymphocytes infiltrated into the lungs following adenoviral infection were important to, through the release of IFNγ, confer a primed immunity phenotype to AMs, resulting in robust host resistance against heterologous bacterial infection [29]. Here, MuHV-4 priming of the three mouse strains results in both an expansion of the activated CD8^+^ pool and a PVM resistance phenotype, which suggests a similar dialogue between bystander CD8^+^ T cells and AMs.

Additionally, four weeks after MuHV-4 infection and in the three mouse strains, AMs displayed a new membrane phenotype characterized by overexpression of MHC II (Figure 3C and Figure 5C). BALB/c mice AMs examination suggested a monocytic (SiglecF^lo^) origin in which AMs are of embryonic origin and self-sustained in steady state but are maintained with the contribution from blood monocytes during inflammation [15,43,44,45]. On the other hand, CD-1 and C57BL/6 mice AMs examination suggested a lung residential (embryonic, SiglecF^hi^) origin in which AMs are maintained independently of monocytes under inflammatory conditions [29,30]. In CD-1 and C57BL/6 mice, MuHV-4-priming did not alter the size of the lung AM population. SiglecF expression on MuHV-4-primed MHC II^hi^ AMs was unimodally distributed and was as comparatively high as on PBS and _hi_MuHV-4-primed MHC II^lo^ AMs (Figure 3C). To question the possible independence of MHC II^hi^ AMs on circulating monocytes, we used CCR2^−/−^ C57BL/6 mice lacking classical Ly6C^hi^ monocytes, a major contributor to tissue monocyte-derived macrophages [46]. In the absence of such monocytes, compared to MuHV-4 primed wildtype C57BL/6 mice, primed CCR2^−/−^ mice displayed unimpaired ability to upregulate MHC II expression. Altogether, the data thus suggest a lung residential, as opposed to monocytic, origin of MHC II^hi^ AMs after MuHV-4 priming in C57BL/6 mice. In BALB/c mice, on the contrary, MuHV-4 priming resulted in an expansion of the AM population, and the distribution of SiglecF fluorescences among MHC II^hi^ AMs was bimodal (Figure S13), suggesting a dual origin of MHC II^hi^ AMs, both residential and monocytic. To explain these diverging results depending on the mouse strain studied, it was previously demonstrated that the AMs cells depletion/persistence hypotheses also depended on the genetic background of the mouse and the conditions of infection. Indeed, influenza infection in BALB/c mice leads to a significant deterioration in the AMs number in an IFNγ-dependent manner, while the number of the infected C57BL/6 counterparts is not altered, but their phenotype is amended [47]. These results are themselves corroborated by several reports demonstrating depletion of AMs in BALB/c mice [48] and a sustained presence of AMs in C57BL/6 mice [49,50,51,52]. Therefore, future analyses of CCR2-depleted BALB/c mice might interrogate distinct mechanisms of MuHV-4-induced suppression of AMs, which could also highlight beneficial or detrimental effects in front of viral heterologous infections. This novel information will be of crucial importance for choosing appropriate mouse models for future experiments.

Modification of the AMs’ IL-6 response by prior exposure to MuHV-4 could also be a key event. Indeed, IL-6 has been associated with severe clinical forms of human pneumovirus (hRSV) [53,54], pandemic H1N1 influenza virus [55], or H5N1 influenza A virus [56] infections. Similarly, in mice, lung IL-6 concentrations were correlated with the severity of symptoms and lesions [57]. Here, we have shown that the most severely affected mice (BALB/c) also produce by far the most pulmonary IL-6 (Figure 4C). This result is of crucial importance considering that, and in contrast to influenza A viruses [58,59], PVM has been significantly less lethal in IL-6^−/−^ mice than in WT mice, an observation corroborated by less neutrophil recruitment and less fluid infiltration in the lungs [60]. Taken together, these results suggest that the magnitude of the IL-6 response controls the severity of pneumoviral disease. Here, the combination of the initial kinetics measured ex vivo and the snapshot measured at day 6 post infection reveals an aspect not previously detected. It is not the amount of pulmonary IL-6 during disease that correlates with severity, but most likely the timing of its secretion by AMs. Similarly, it was demonstrated that AMs depletion prior to PVM infection results in prolonged survival with small but statistically significant increases in virus recovery [61]. Additionally, because neither patrolling monocytes (Ly6C^lo^) nor inflammatory monocytes (Ly6C^hi^) but AMs are the primary source of IL-6 in the experimental disease induced by PVM inoculation to mice [60,61], the ex vivo comparison of the AMs response in a longitudinal design is representative of the IL-6 response in vivo. Thus, an AM-subordinated, potent, immediate, and rapidly suppressed IL-6 outburst is associated with mild disease and, conversely, a moderate, delayed, and prolonged response is associated with severe disease.

Additionally, excessive TNF-α production during some respiratory viral infections has also been associated with excessive production of cytokines, lung destruction, immunopathology, and premature death [62,63,64]. Further, TNF-α deficiency or immunoneutralization improved survival and lung pathology without affecting viral loads [62], which is reminiscent of our results. As with IL-6, the kinetics of TNF-α production was different in MuHV-4 primed mice, with the generation of an immediate, high-magnitude outburst that is absent in mock-primed mice upon exposure to PVM, followed by a delayed attenuation. While there is little doubt that TNF-α participates in the PVM disease severity as in other viral infections, the beneficial effect of priming with MuHV-4 reveals that kinetics plays a much more important role than anticipated, which may explain why constitutive TNF deficiency is just as deleterious as excess production in respiratory viral diseases [64].

On the other hand, we demonstrate that activated AMs are poised for robust primed immunity upon ex vivo bacterial stimulation via rapid induction of neutrophil chemokines CXCL1 and CXCL2, providing, therefore, enhanced antibacterial protection. Our results confirm previous publication suggesting that memory AMs, but not other macrophage subsets, in the lung of viral-exposed animals mediate antibacterial trained immunity via similar enhanced neutrophil chemokine production [29]. Additionally, in mock-primed mice, the degree of neutrophilic infiltration and the increased expression of chemokines and neutrophil activators, especially CXCL1 and CXCL2, into the lungs following PVM inoculation has been positively correlated with the severity of hRSV-induced bronchiolitis [65,66,67,68,69]. As the more rapid attenuation of CXCL1 and CXCL2 cytokine production in MuHV-4-primed mice likely plays a role in the drastic decrease in neutrophil infiltration in the lungs measured 6 days p.i. (Figure 3B), it, therefore, participates at least in part in the anti-pneumoviral protection conferred by prior exposure to MuHV-4. Similar experiments demonstrated that neutralization of CXCR2, the receptor for the chemokines CXCL1, CXCL2, granulocyte chemoattract protein 2, and LPS-induced CXC chemokine, prevents RSV-induced airway hyperreactivity but does not alter viral clearance [70]. These results further support a role for CXCL1 and CXCL2 in the induction of hRSV-associated immunopathologic mechanisms and suggest that the rapid attenuation of these chemokines might allow anti-pneumoviral protection by targeting the lethal inflammatory sequelae of PVM infection.

Finally, we demonstrated that, upon ex vivo bacterial or viral stimulation, MuHV-4-imprinted AMs from CCR2^−/−^ mice showed a qualitatively similar response to that of imprinted AMs from wildtype mice. Quantitatively, however, the magnitude of the early IL-6, CXCL1, CXCL2, TNF-α, and CCL3 responses was much lower (Figure 6C). These observations suggest that the final maturation of MuHV-4-dependent imprinting, thus the full deployment of the anti-PVM protection conferred by prior MuHV-4 infection, requires the infiltration of inflammatory monocytes into the lungs prior to exposure to PVM.

## 5. Conclusions

Taken together, our results showed that prior respiratory infection of the laboratory mouse with a gammaherpesvirus prepares resident AMs to respond differently to subsequent exposure to a pneumovirus. This immune priming consists of modifying their immediate cyto/chemokine responses upon exposure, thereby drastically attenuating the host inflammatory response without affecting viral replication. Moreover, even if we have demonstrated that primed AMs do not result from monocytes origin, these latter remain necessary for the establishment of an improved immune response following MuHV-4 priming by an important modification of the AMs cyto/chemokine response. The role of monocytes in this mechanism should therefore be analyzed in the future to determine which cellular interactions with AMs lead to improved innate immunity.

## Data Availability

The data presented in this study are available in the article and are available on reasonable request from the corresponding author.

## Supplementary Materials

The following are available online at https://www.mdpi.com/article/10.3390/v14010098/s1, Table S1: Recap table of leukocytes subpopulations modifications compared to naïve mice lungs, Figure S1: Gating strategy developed to discriminate lung myeloid cell subsets using flow cytometry, Figure S2: Gating strategy developed to discriminate lung lymphoid cell subsets, Figure S3: Gating strategy developed to discriminate lung basophils, mast cells and plasmacytoid dendritic cells cell subsets, Figure S4: Fluorescence-minus-one controls vs. full panel fluorescence for myeloid adequate gating strategy, Figure S5: Fluorescence-minus-one controls vs. full panel fluorescence for lymphoid adequate gating strategy, Figure S6: Comparison of strain and priming effects on mouse survival after increasing doses of PVM were inoculated, Figure S7: Past MuHV-4 infection decreases morbidity and attenuates lung weight gains upon subsequent PVM disease, Figure S8: MuHV-4 priming adjusts lung lymphoid subpopulations both before and 6 days after exposure to PVM, Figure S9: Lung monocytic subpopulations both before and 6 days after exposure to PVM, Figure S10: Lung granulocytic subpopulations both before and 6 days after exposure to PVM, Figure S11: Past MuHV-4 infection adjusts lung cyto- and chemokine responses upon subsequent PVM disease, Figure S12: Density plot showing the presence or, conversely, the absence of Ly6C^high^/CD192^high^ infiltrated monocytes in the lungs of PBS- and MuHV-4-primed wildtype or CCR2^−/−^ C57BL/6 mice, respectively, Figure S13: Past MuHV-4 infection differently modify AMs phenotype in BALB/c compared to CD-1 and C57BL/6 mice.
