# Supplementary material for: Gammaherpesvirus Alters Alveolar Macrophages According to the Host Genetic Background and Promotes Beneficial Inflammatory Control over Pneumovirus Infection
Source: Viruses. 2022 Jan 6;14(1):98. doi: 10.3390/v14010098 (PMC8777918; doi:10.3390/v14010098)
Supplement: Supplementary file 1 [file viruses-14-00098-s001.zip › viruses-1520262-supplementary.pdf]

viruses

MDPI

Supplementary Materials

Gammaherpesvirus Alters Alveolar Macrophages According to the Host Genetic Background and Promotes Beneficial Inflammatory Control over Pneumovirus Infection

Table S1: Recap table of infectious groups and associated leukocytes subpopulations modifications compared to naïve mice lungs.

Mice strains	Leukocytes	PVM	MuHV-4	MuHV-4 + PVM
BALB/c	iNKT CD4-	↗ ***	-	↗ ***
	iNKT CD4+	↗ ***	-	↗ ***
	NK Cells	↗ ***	-	↗ ***
	B Cells	-	-	-
	CD4+ T Cells	-	-	-
	CD8+ T Cells	-	↗ *	↗ ***
	γδ T Cells	-	↗ *	↗ ***
	DN T Cells	-	-	↗ ***
	AM	↗ ***	↗ ***	↗ ***
	nAM	-	-	-
	Ly6C Low Mo	↗ **	-	↗ ***
	Ly6C High Mo	↗ *	-	↗ **
	CD11b+ DC	↗ **	-	↗ ***
	CD103+ DC	-	↗ *	↗ **
	pDC	↗ ***	-	↗ ***
	Eosinophils	↗ ***	-	↗ ***
	Neutrophils	↗ ***	-	-
	Basophils	↗ ***	-	↗ ***
	Mast Cells	↗ ***	-	↗ ***
CD-1	iNKT CD4-	↗ ***	↗ **	↗ ***
	iNKT CD4+	↗ ***	-	↗ ***
	NK Cells	↗ ***	-	↗ ***
	B Cells	-	↗ *	-
	CD4+ T Cells	-	-	-
	CD8+ T Cells	-	-	↗ ***
	γδ T Cells	-	-	-
	DN T Cells	-	-	↗ ***
	AM	↗ ***	-	↗ *
	nAM	↗ **	↗ ***	↗ ***
	Ly6C Low Mo	-	-	-
	Ly6C High Mo	↗ ***	-	↗ *
	CD11b+ DC	-	-	↗ **
	CD103+ DC	↗ ***	-	↗ ***
	pDC	↗ ***	-	↗ ***
	Eosinophils	↗ ***	-	↗ **
	Neutrophils	↗ ***	-	↗ ***
	Basophils	-	-	-
	Mast Cells	-	-	-
C57BL/6	iNKT CD4-	↗ ***	↗ ***	-

	iNKT CD4+	↗ **	-	↗ ***
	NK Cells	↗ ***	↗ **	↗ ***
	B Cells	-	↗ ***	↗ ***
	CD4+ T Cells	-	↗ **	↗ ***
	CD8+ T Cells	-	↗ ***	↗ ***
	γδ T Cells	-	-	-
	DN T Cells	-	-	↗ ***
	AM	↗ ***	-	↗ **
	nAM	-	-	↗ **
	Ly6C Low Mo	↗ ***	-	-
	Ly6C High Mo	-	-	-
	CD11b+ DC	↗ *	-	↗ ***
	CD103+ DC	-	-	↗ **
	pDC	↗ ***	-	↗ ***
	Eosinophils	↗ ***	-	↗ ***
	Neutrophils	↗ **	-	↗ **
	Basophils	↗ *	-	↗ ***
	Mast Cells	↗ ***	-	↗ ***

"↗" = Increase with *$p < 0.05$, **$p < 0.01$ or ***$p < 0.001$; "-"= no significant modifications. PVM: pneumonia virus of mice; MuHV-4: murid herpesvirus 4; iNKT: invariant natural killer T cells; NK: natural killer; DN T cells: double negative T cells; AM: alveolar macrophages; nAM: non-alveolar macrophages; Mo: monocytes; DC: Dendritic cells; pDC: plasmacytoid dendritic cells.

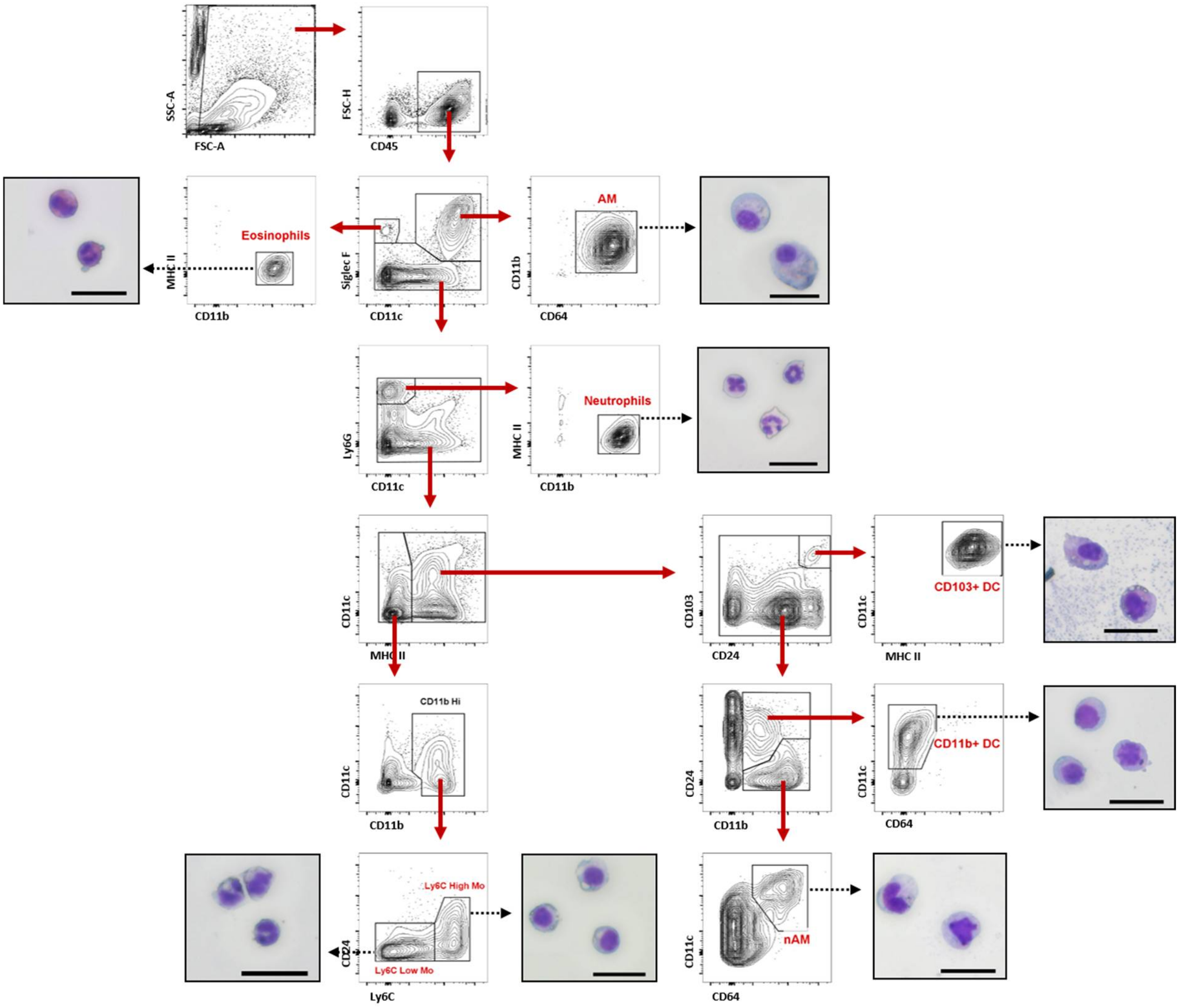

Figure S1. Gating strategy developed to discriminate lung myeloid cell subsets using flow cytometry. After exclusion of debris and doublets (not shown), immune cells were identified using the pan-hematopoietic marker CD45. AMs (SiglecF$^+$, CD11c$^+$, CD64$^+$, CD11b$^-$), eosinophils (SiglecF$^+$, CD11c$^-$, CD11b$^+$) and neutrophils (Ly6G$^+$, CD11c$^-$) were readily identified first. After, low expression of MHC II permitted the separation of monocytes (CD11b$^+$, MHC II$^-$) from non-alveolar macrophages (nAMs) (CD11b$^+$, MHC II$^+$, CD24$^-$), CD103$^+$DC (CD11b$^-$, MHC II$^+$, CD103$^+$) and CD11b$^+$DC (CD11b$^+$, MHC II$^+$, CD64$^-$) subsets. Besides, subdivision between “inflammatory” and “stationary” monocytes was completed based on the Ly6C level of expression. Scale bar on microphotographs: 20 μm.

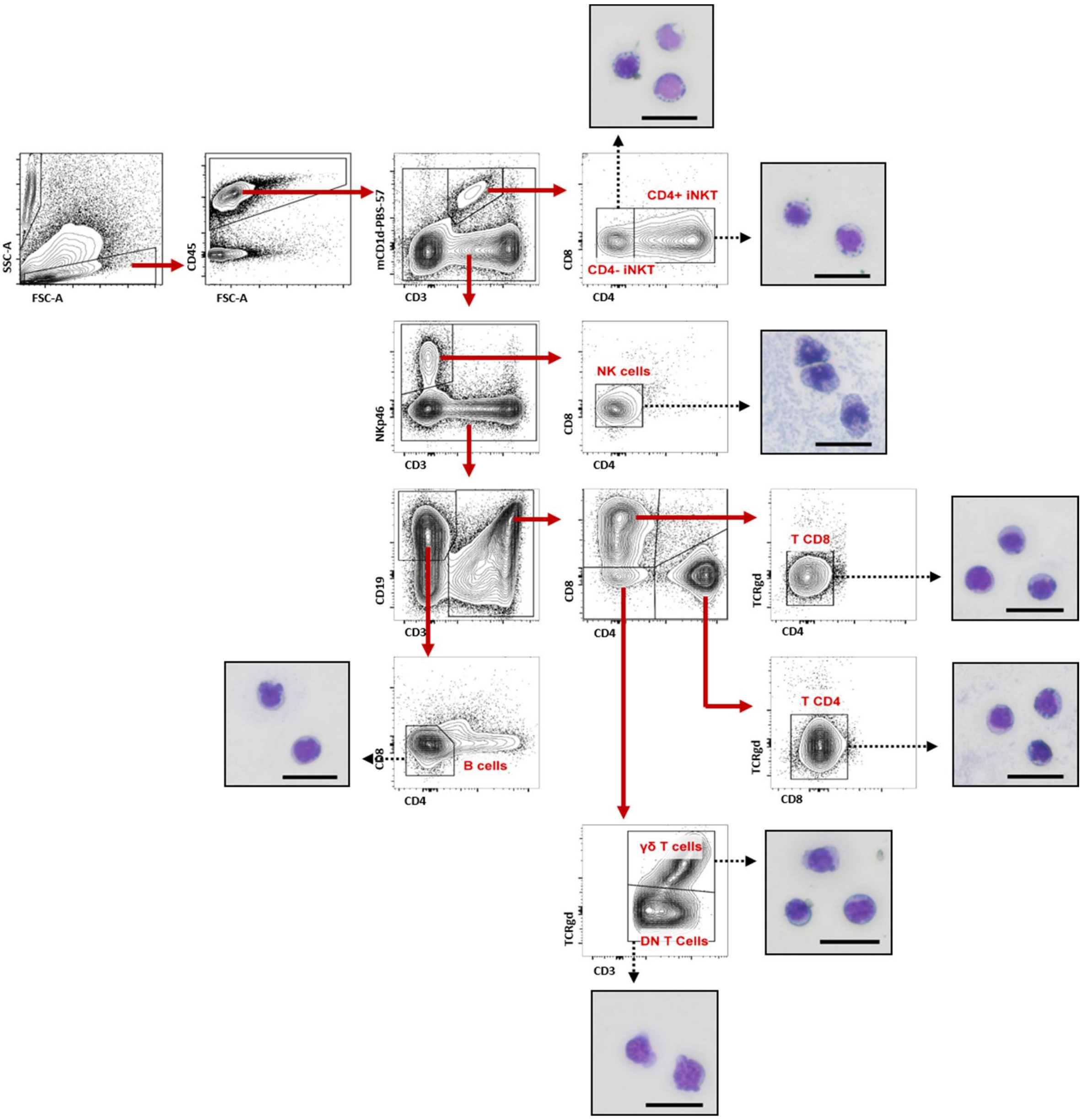

Figure S2. Gating strategy developed to discriminate lung lymphoid cell subsets. After exclusion of debris and doublets (not shown), immune cells were identified using the pan-hematopoietic marker CD45. iNKT cells (mCD1d-PBS-57$^+$), themselves subdivided into CD4 positive or negative iNKT cells, and NK cells (CD3$^-$, NKp46$^+$) were readily identified. B cells (CD3$^-$, CD19$^+$) were isolated from T cells subsets (CD3$^+$, CD19$^-$). Among the CD3$^+$ T cells, the CD4$^+$ (CD3$^+$, CD4$^+$, CD8$^-$) and CD8$^+$ (CD3$^+$, CD4$^-$, CD8$^+$) T cells then were gated according to staining with the corresponding mAbs. Finally, among the remaining CD3$^+$ T cells, the γδ T cells (CD3$^+$, CD4$^-$, CD8$^-$, γδ TCR$^+$) and the DN T cells (CD3$^+$, CD4$^-$, CD8$^-$, γδ TCR$^-$) were identified. Scale bar on microphotographs: 20 μm.

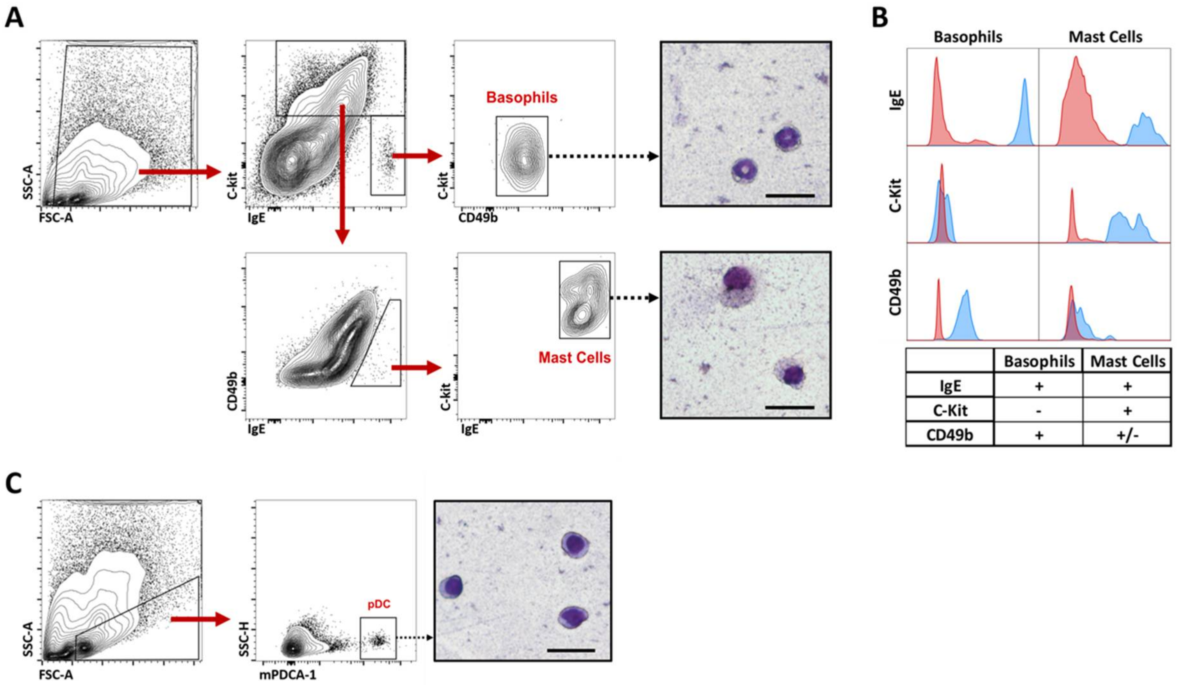

Figure S3. Gating strategy developed to discriminate lung basophils, mast cells and plasmacytoid dendritic cells cell subsets. After exclusion of debris and doublets (not shown), a sequential gating strategy was employed to identify specific cell populations based on their expression of a cocktail of markers. (**A**) Basophils (IgE^{+}, C-kit^{-}, CD49b^{+}) and mast cells (IgE^{+}, C-kit^{+}, CD49b$^{+/-}$) were identified according to their c-kit expressing level. (**B**) Expression of the various markers used to discriminate the basophils from mast cells. Red: FMO control fluorescence; blue: full panel fluorescence. +, high expression; +/–, low or intermediate expression and –, absence of expression. (**C**) pDCs were easily identified by their specific mPDCA-1- positivity. Scale bar on microphotographs: 20 µm.

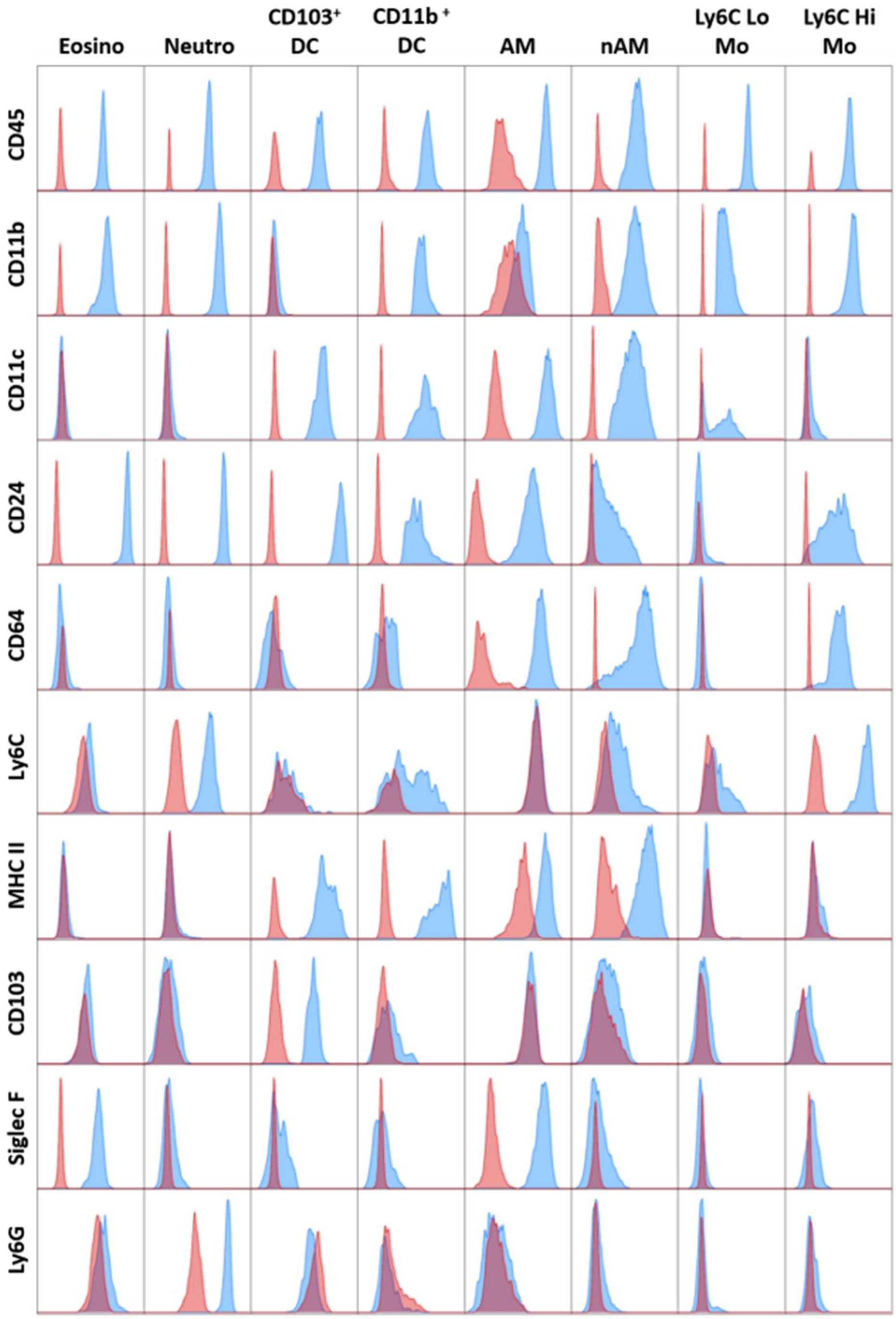

	Eosinophils	Neutrophils	CD103+ DC	CD11b+ DC	AM	nAM	Ly6C Lo Mo	Ly6C Hi Mo
CD45	+	+	+	+	+	+	+	+
CD11b	+	+	-	+	-	+	+	+
CD11c	-	-	+	+	+	+	+/-	-
CD24	+	+	+	+	+	+/-	-	+/-
CD64	-	-	-	-	+	+/-	-	+/-
Ly6C	-	+	-	+/-	-	+/-	+/-	+
MHC II	-	-	+	+	+	+	-	-
CD103	-	-	+	-	-	-	-	-
Siglec F	+	-	-	-	+	-	-	-
Ly6G	-	+	-	-	-	-	-	-

Figure S4. Fluorescence minus one controls vs. full panel fluorescence for myeloid adequate gating strategy. FMO controls were used on single cell suspensions of digested mouse lung and analyzed by flow cytometry. This figure shows how the gate for the specific subsets (Figure S1) was set as compared to the different FMO controls. Red histograms: FMO control fluorescence; Blue

histograms: full panel fluorescence. +, high expression; +/–, low or intermediate expression and –, absence of expression.

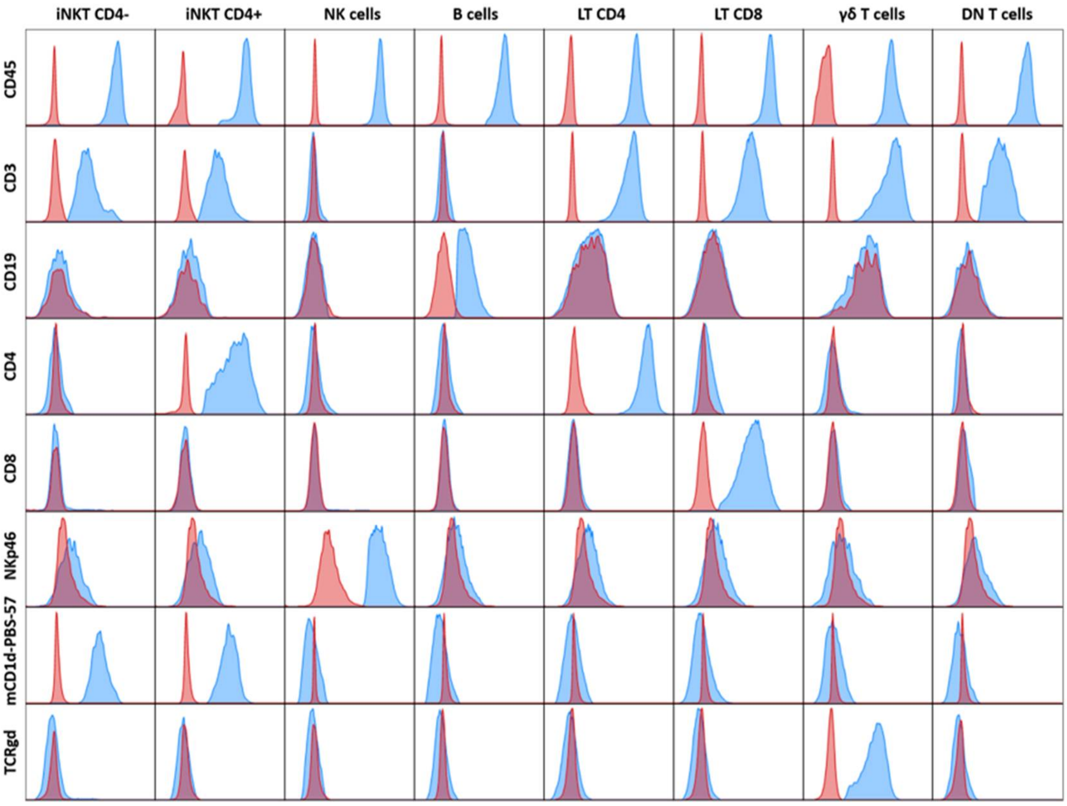

	iNKT CD4-	iNKT CD4+	NK cells	B cells	LT CD4	LT CD8	γδ T cells	DN Tcells
CD45	+	+	+	+	+	+	+	+
CD3	+	+	-	-	+	+	+	+
CD19	-	-	-	+	-	-	-	-
CD4	-	+	-	-	+	-	-	-
CD8	-	-	-	-	-	+	-	-
NKp46	-	-	+	-	-	-	-	-
mCD1d-PBS-57	+	+	-	-	-	-	-	-
TCRgd	-	-	-	-	-	-	+	-

Figure S5. Fluorescence minus one controls vs. full panel fluorescence for lymphoid adequate gating strategy. FMO controls were used on single cell suspensions of digested mouse lung and analyzed by flow cytometry. This figure shows how the gate for the specific subsets (Figure S2) was set as compared to the different FMO controls. Red histograms: FMO control fluorescence; Blue histograms: full panel fluorescence. +, high expression; +/–, low or intermediate expression and –, absence of expression.

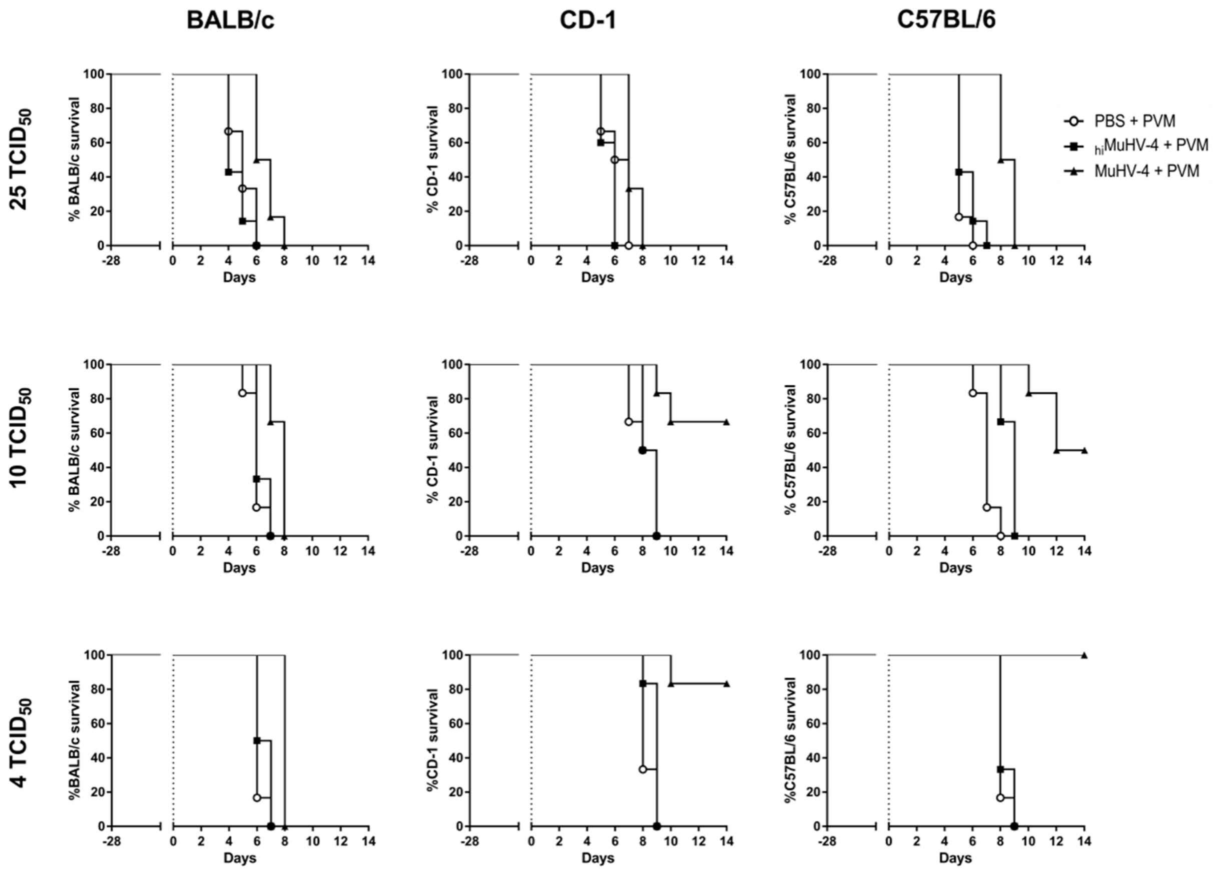

Figure S6. Comparison of strain and priming effects on mouse survival after increasing doses of PVM were inoculated. Twenty-eight days before PVM inoculation, CD-1, BALB/c and C57BL/6 mice had been inoculated intranasally with 1.4 × 10^4 $TCID_{50}$ MuHV-4, $_{hi}$MuHV-4, or PBS. On day 0, all mice were infected intranasally with PVM (25, 10 or 4 $TCID_{50}$ units) and the survivability of each mice strain was monitored. (*n* = 6 mice/mouse strain/priming condition; representative of three independent experiments). See text for key.

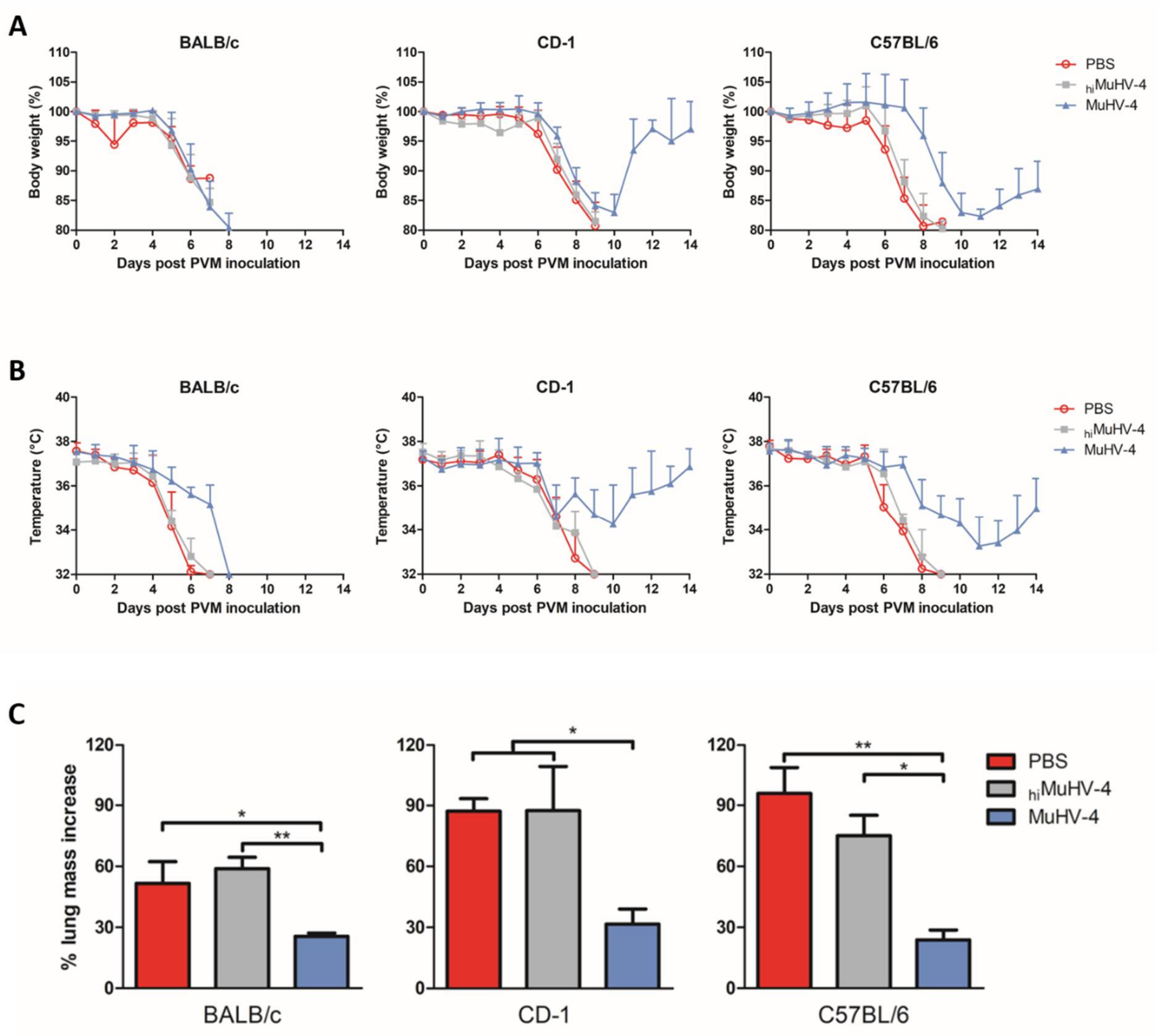

Figure S7. Past MuHV-4 infection decreases morbidity and attenuates lung weight gains upon subsequent PVM disease. Body weight (**A**) and temperature (**B**) follow-up after PVM inoculation (4 $TCID_{50}$ units), according to strain and priming format (n = 6 mice/group). 100 % bodyweight correspond to the initial weight of the animal. **(C)** histograms representation of the relative weight gain of the lungs 6 days after PVM inoculation compared to the lung weights of uninfected strain-, sex- and age-matched mice. The weight gain is expected to correlate with magnitude of accumulated cells, blood and edema within diseased lungs. All results are reported as mean ± SD, with n = 6 mice/mouse strain/priming condition; representative of three independent experiments. Significantly different means are highlighted, with *p < 0.05, **p < 0.01 or ***p < 0.001. n.s., not significant (one-way ANOVA and Bonferroni post-tests). See text for key.

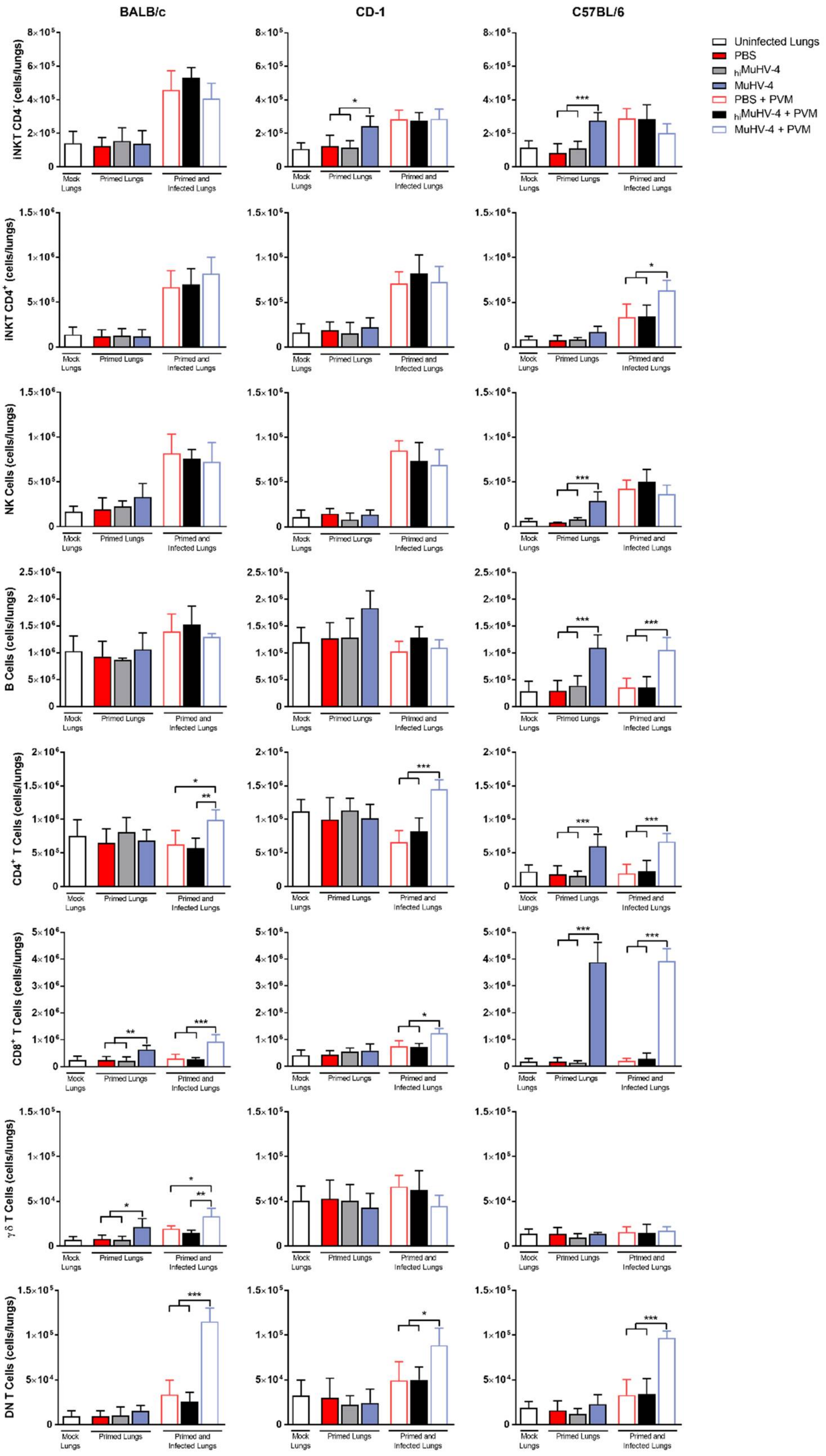

Figure S8. MuHV-4 priming adjusts lung lymphoid subpopulations both before and 6 days after exposure to PVM. Enumeration of absolute lung lymphoid subpopulations in control (mock), primed and primed/infected CD-1, BALB/c and C57BL/6 lungs (mean ± SD, with n = 6 mice/mouse strain/priming condition; representative of three independent experiments). Mock lungs, primed lungs (day 0) and primed/infected lungs (day 6) are considered separately from each other for

statistical analysis. Significantly different means are highlighted, with *$p < 0.05$, **$p < 0.01$ or ***$p < 0.001$. n.s., not significant (one-way ANOVA and Bonferroni post-tests).

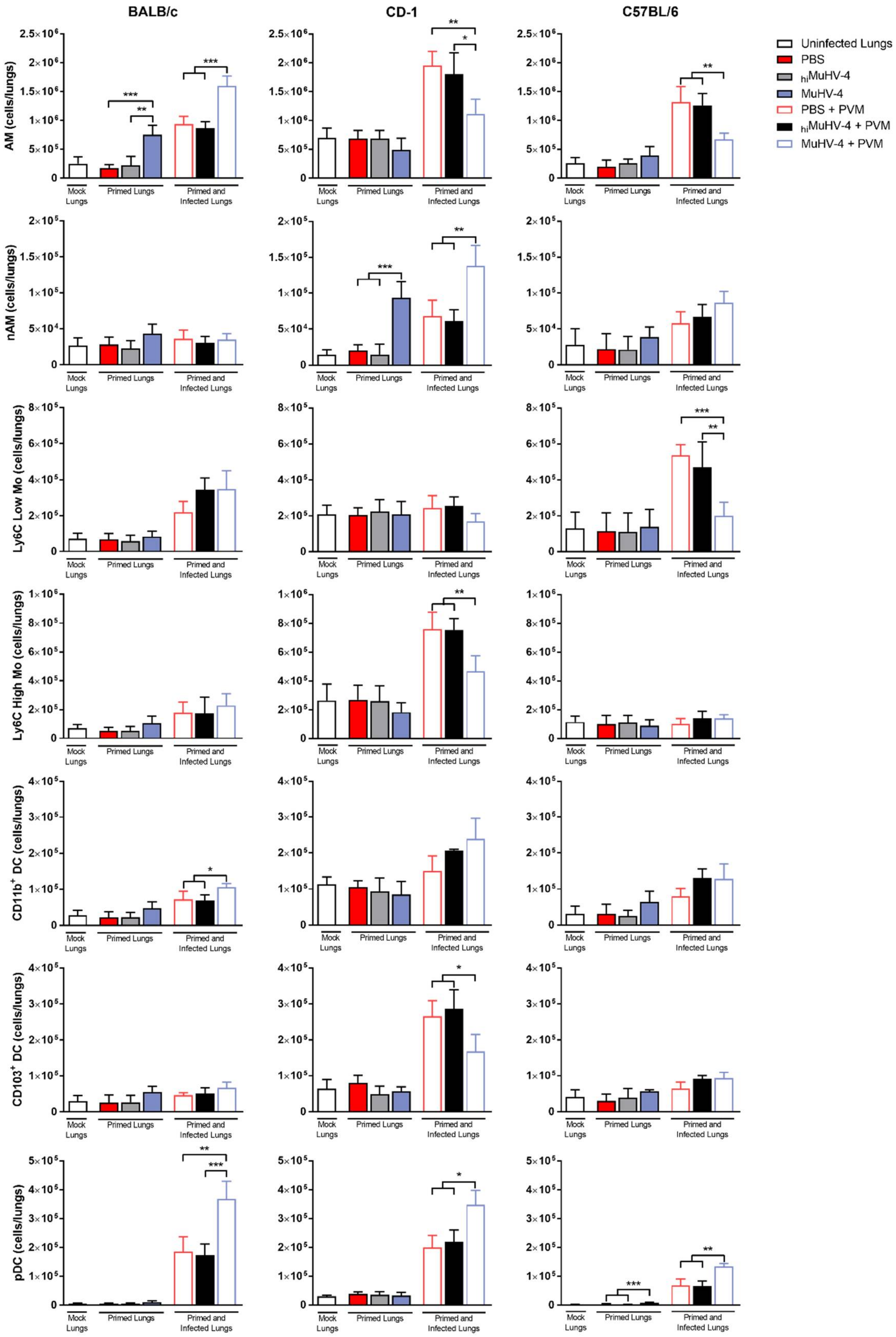

Figure S9. Lung monocytic subpopulations both before and 6 days after exposure to PVM. Enumeration of absolute lung monocytic subpopulations in control (mock), primed and primed/infected CD-1, BALB/c and C57BL/6 lungs (mean ± SD,

with n = 6 mice/mouse strain/priming condition; representative of three independent experiments). Mock lungs, primed lungs (day 0) and primed/infected lungs (day 6) are considered separately from each other for statistical analysis. Significantly different means are highlighted, with *$p < 0.05$, **$p < 0.01$ or ***$p < 0.001$. n.s., not significant (one-way ANOVA and Bonferroni post-tests).

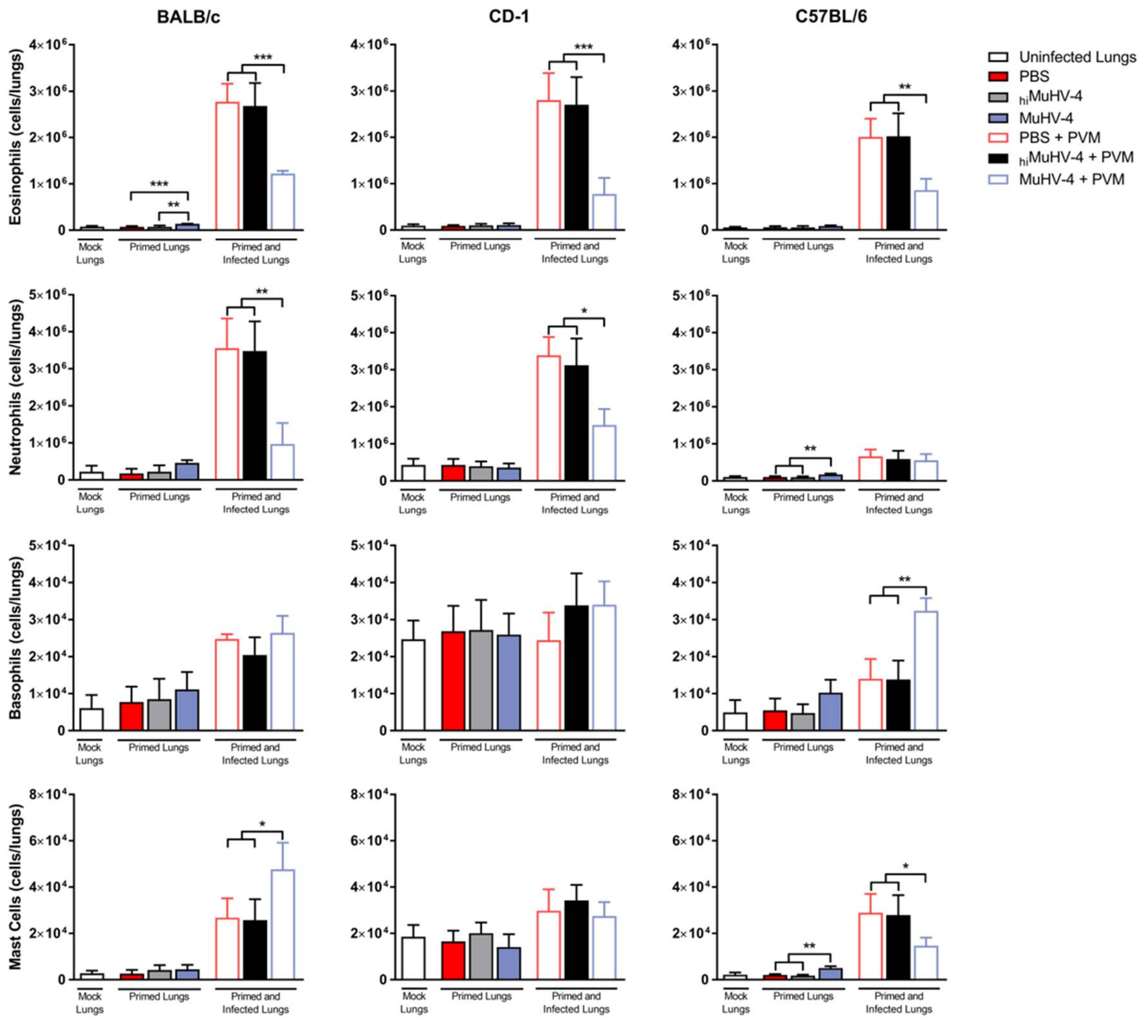

Figure S10. Lung granulocytic subpopulations both before and 6 days after exposure to PVM. Enumeration of absolute lung monocytic subpopulations in control (mock), primed and primed/infected CD-1, BALB/c and C57BL/6 lungs (mean ± SD, with n = 6 mice/mouse strain/priming condition; representative of three independent experiments). Mock lungs, primed lungs (day 0) and primed/infected lungs (day 6) are considered separately from each other for statistical analysis. Significantly different means are highlighted, with *$p < 0.05$, **$p < 0.01$ or ***$p < 0.001$. n.s., not significant (one-way ANOVA and Bonferroni post-tests). See text for key.

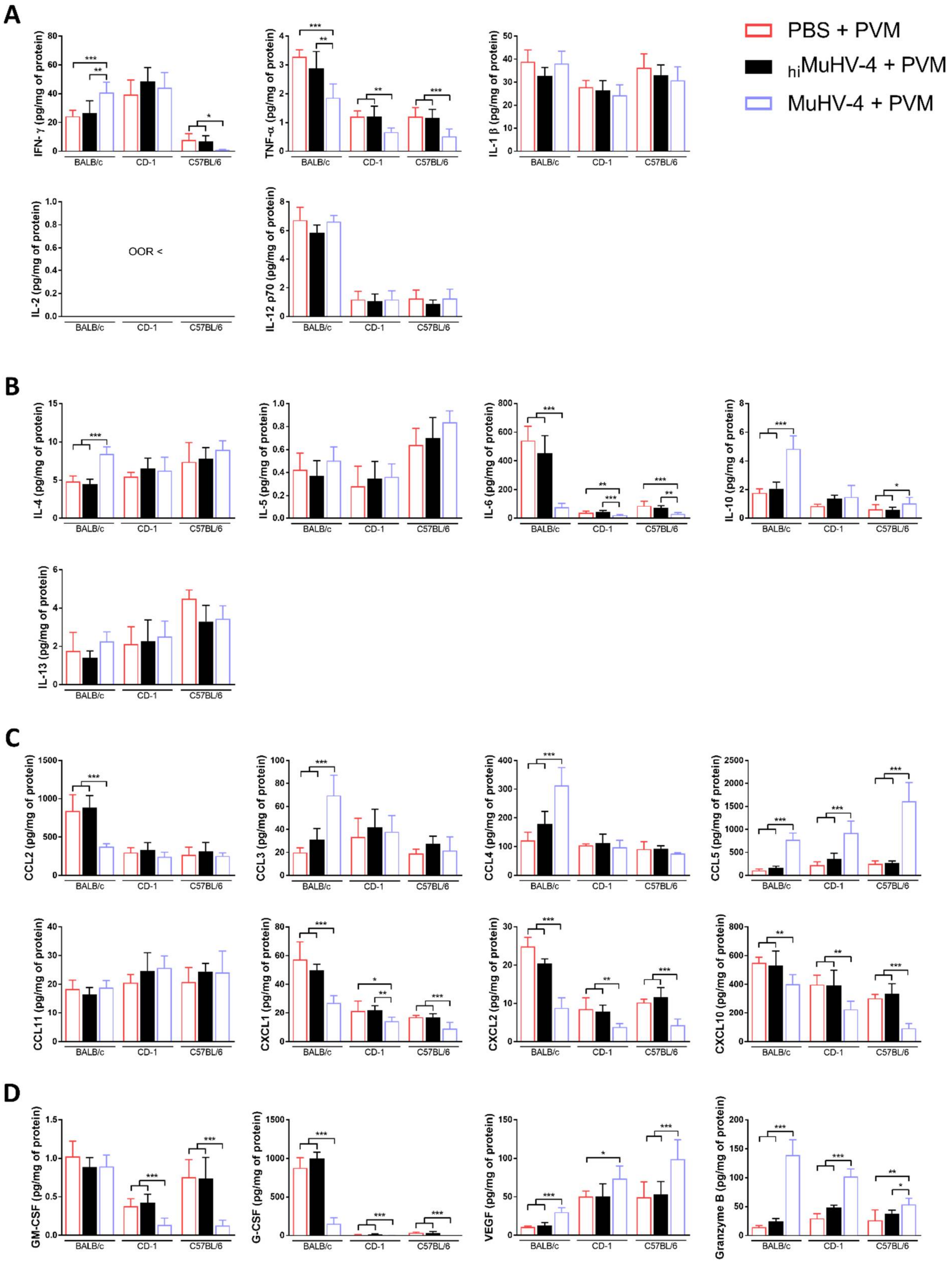

Figure S11. Past MuHV-4 infection adjusts lung cyto- and chemokine responses upon subsequent PVM disease. The histograms show the normalized lung cyto/chemokine concentrations measured in BALB/c, CD-1 and C57BL/6 PBS, $_{hi}$MuHV-4 or MuHV-4-primed mice on day 6 post PVM infection. (**A**) Th1 cytokines. (**B**) Th2 cytokines. (**C**) Chemokines. (**D**) GM-CSF, G-CSF, VEGF and Granzyme B. All results are reported as mean ± SD, with n = 6 mice/mouse strain/priming condition; representative of three independent experiments. Significantly different means are highlighted, with *p < 0.05, **p < 0.01 or ***p < 0.001. n.s., not significant (one-way ANOVA and Bonferroni post-tests). See text for key.

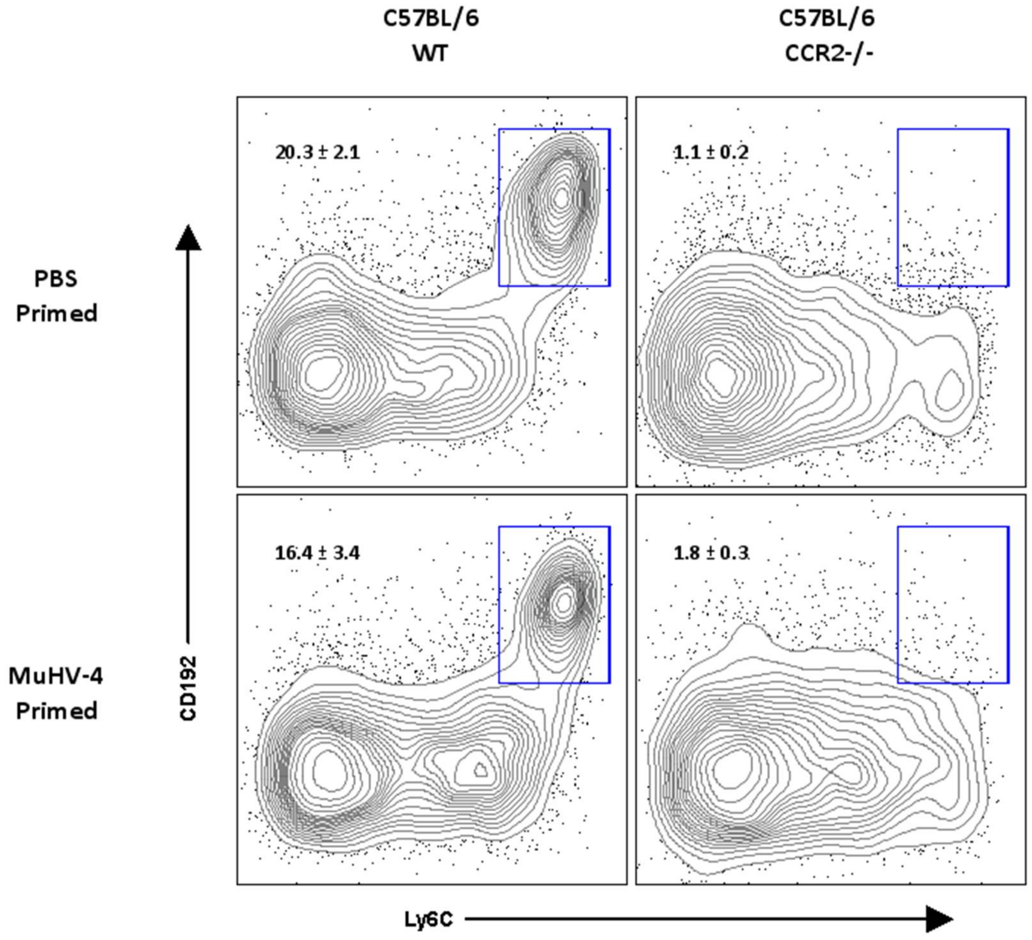

Figure S12. Density plot showing the presence or, conversely, the absence of Ly6C^{high}/CD192^{high} infiltrated monocytes in the lungs of PBS- and MuHV-4-primed wildtype or CCR2$^{-/-}$ C57BL/6 mice, respectively. CCR2 deficiency was confirmed using CD192 antibody and the lacking of classical Ly6C^{hi} monocytes recruitment was confirmed in pulmonary monocyte population. The numbers in each quadrant (mean ± SD) correspond to the relative proportions of CCR2^{+} monocyte sub-populations to the respective total monocyte populations.

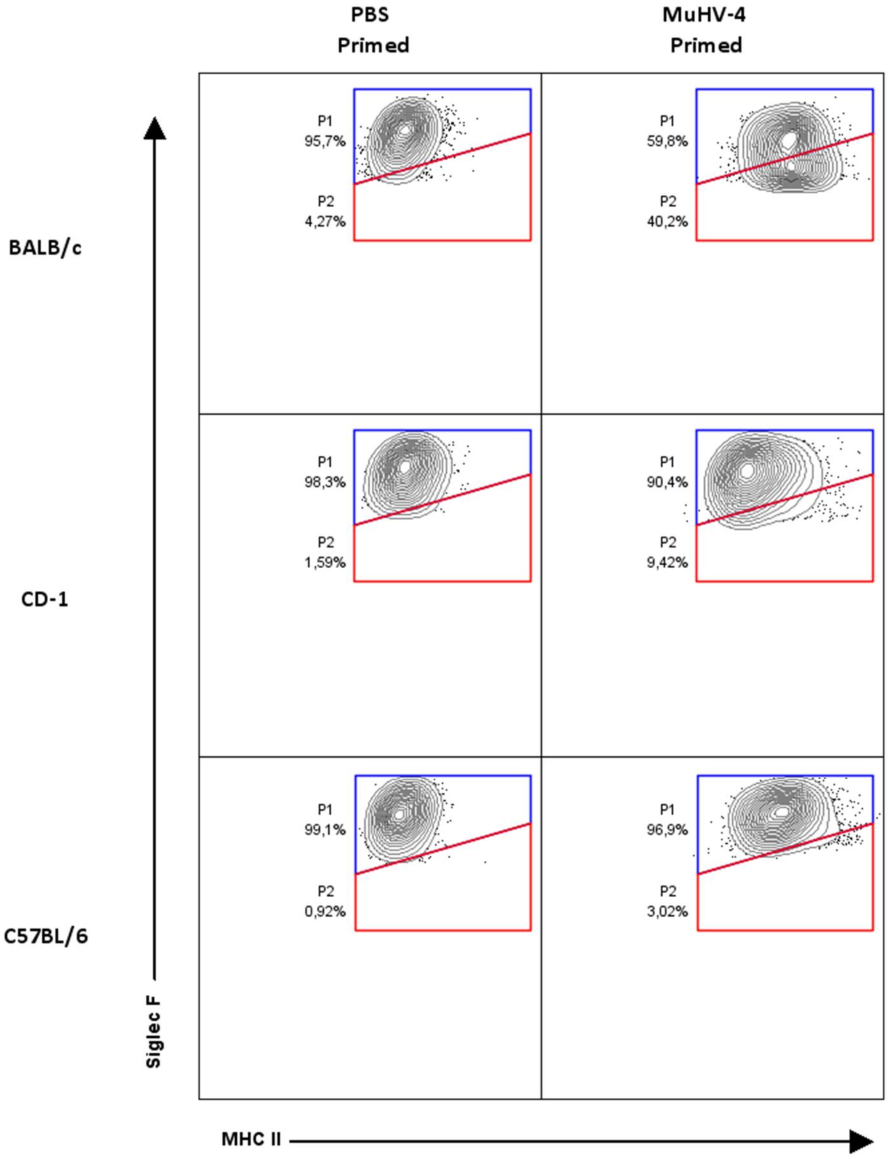

Figure S13. Past MuHV-4 infection differently modify AMs phenotype in BALB/c compared to CD-1 and C57BL/6 mice. The SiglecF/MHC II density plots confirm a shared increased MHC II expression in MuHV-4 primed AMs and a new, BALB/c-specific SiglecF^{low} AM subpopulation. See text for key.
